# The pre-hatching bovine embryo transforms the uterine luminal metabolite composition *in vivo*

**DOI:** 10.1038/s41598-019-44590-9

**Published:** 2019-06-07

**Authors:** Mariana Sponchiado, Angela M. Gonella-Diaza, Cecília C. Rocha, Edson G. Lo Turco, Guilherme Pugliesi, Jo L. M. R. Leroy, Mario Binelli

**Affiliations:** 1 Department of Animal Reproduction, School of Veterinary Medicine and Animal Science, University of São Paulo, Pirassununga, SP Brazil; 2 Gamete Research Centre, Faculty of Biomedical, Pharmaceutical and Veterinary Sciences, University of Antwerp, Antwerp, Belgium; 3 North Florida Research and Education Center, Institute of Food and Agricultural Sciences, University of Florida, Marianna, FL USA; 4 Human Reproduction Section, Division of Urology, Department of Surgery, São Paulo Federal University, Sao Paulo, SP Brazil; 5 Department of Animal Sciences, University of Florida, Gainesville, FL USA

**Keywords:** Infertility, Diagnostic markers

## Abstract

In cattle, conceptus development after elongation relies on well-characterized, paracrine interactions with the hosting maternal reproductive tract. However, it was unrecognized previously that the pre-hatching, pre-implantation bovine embryo also engages in biochemical signalling with the maternal uterus. Our recent work showed that the embryo modified the endometrial transcriptome *in vivo*. Here, we hypothesized that the embryo modulates the biochemical composition of the uterine luminal fluid (ULF) in the most cranial portion of the uterine horn ipsilateral to the corpus luteum. Endometrial samples and ULF were collected post-mortem from sham-inseminated cows and from cows inseminated and detected pregnant 7 days after oestrus. We used quantitative mass spectrometry to demonstrate that the pre-hatching embryo changes ULF composition *in vivo*. Embryo-induced modulation included an increase in concentrations of lipoxygenase-derived metabolites [12(S)-HETE, 15(S)-HETE] and a decrease in the concentrations of amino acids (glycine), biogenic amines (sarcosine), acylcarnitines and phospholipids. The changed composition of the ULF could be due to secretion or depletion of specific molecules, executed by either the embryo or the endometrium, but initiated by signals coming from the embryo. This study provides the basis for further understanding embryo-initiated modulation of the uterine milieu. Early embryonic signalling may be necessary to guarantee optimal development and successful establishment of pregnancy in cattle.

## Introduction

In cattle, the embryo transits from the oviduct to the uterine lumen and remains loosely attached during the 20-days pre-implantation period. This is a critical window for pregnancy wherein as much as 40% of embryos die^1^. After implantation, embryo mortality decreases as hemotrophic nutrition is accomplished through placentation. Causes of mortality that occur before implantation are likely associated with disruptions on the complex biochemical interactions that take place between the developing conceptus (embryo and associated membranes) and the endometrium. Interactions between the endometrium and the conceptus occur through the exchange of secretions from both units into the uterine lumen. Secretions that originate from the endometrium are called the histotroph. The histotroph is composed of hormone-mediated, selective transudation of plasma components and release of locally *de novo* synthesized molecules that reach the uterine lumen through excretion from endometrial glands and transport across the epithelium lining the endometrium^2^. The arrival of the embryo into the uterus adds molecular complexity to this scenario, as the embryo releases additional molecules into the uterine luminal fluid (ULF). Moreover, molecules originating from each unit have the potential to influence the function of each other. The classical example is the effect of conceptus derived interferon-tau that inhibits prominent pulses of prostaglandin-F2alpha from the endometrium in cattle^3^. Furthermore, both the conceptus and the endometrium likely utilize molecules present in the ULF to support cellular proliferation and function. Finally, it is expected that both maternal and embryonic influences on the uterine environment composition change as the pregnancy progresses towards implantation. In summary, the biochemical composition of the ULF dynamically reflects the contributions and the consumption of molecules by both the maternal and the embryonic units during the pre-implantation window.

Exposure to histotroph is a prerequisite for development of the embryo after the hatched blastocyst stage *in vivo*. Indeed, efforts to artificially induce elongation of bovine conceptuses *in vitro* have been unsuccessful^4,5^. Furthermore, perturbations of histotroph composition prior to embryo transfer severely impaired embryo survival and pregnancy establishment in cattle^6^. However, specific luminal metabolite requirements of the earliest phase of embryo development in the uterus are unknown. Nature and concentration of specific molecules likely reflect the changing requirements of the developing embryo in response to a changing nutrient supply during its migration from the oviduct to the uterus and, subsequently, along the uterine lumen.

Exposure of the endometrium to ULF conditioned by the elongated conceptus is required for the maintenance of pregnancy^7^. Conceptus-originated molecules, such as interferon-tau and prostaglandins, re-program function of endometrial cells from luteolytic to pregnancy-supporting. However, the influence of the pre-elongation embryo on endometrial function is poorly understood. *In vitro* studies have shown that pre-implantation embryos release a variety of biochemical signals, referred to as embryotropins^8^, that act in concert to support embryonic development. The paracrine effects of embryo-derived molecules on the maternal tissue are expected to be limited to the immediate embryo surroundings. This may be attributed to the capacity of synthesis, secretion and diffusion of signalling molecules by the early-embryo, which is expected to be proportional to its cellular machinery (∼120 cells at the blastocyst stage). Notwithstanding, we reported previously that the endometrial abundance of specific transcripts was altered by the presence of a day-7 embryo in a spatial-specific manner^9^. The most pronounced effects were found in the cranial region of the uterine horn ipsilateral to the corpus luteum (CL), where the embryos were located on day 7. The main pathways changed by the embryo included type I interferon-response and genes associated to the prostaglandin metabolism. In agreement, recent *in vitro* studies showed that early bovine embryos were able to modulate gene expression of co-cultured endometrial^10–12^, oviductal^13^, luteal^14^ and immune cells^10,15,16^. However, a critical unanswered question is whether the female tract has the ability to respond to pre-hatching embryo-derived signals beyond the transcription level, to change its transport and secretory functions and ultimately change the composition of the uterine microenvironment.

We hypothesized that the presence of an embryo modulates the biochemical composition of the ULF in the cranial region of the ipsilateral uterine horn. The aim was to assess a spatially-defined region of the uterine luminal environment, at a time-point coinciding with the apical location of the embryo, to compare the concentration of selected metabolites in ULF between pregnant and sham-inseminated cows. More specifically, we aimed to measure the absolute concentrations of targeted metabolites based on their possible role on early pregnancy biology. The analytes panel included amino acids, biogenic amines, acylcarnitines, lipids, hexoses, and eicosanoids and oxidation products of polyunsaturated fatty acids. Transcripts analyses were performed on endometrial samples to link the findings at the ULF level to the surrounding endometrial tissue.

## Material and Methods

All experimental procedures were performed in accordance with ethical principles in animal research. Protocol was approved by the Ethics and Animal Handling Committee of the School of Veterinary Medicine and Animal Science of the University of São Paulo (CEUA-FMVZ/USP, n3167260815).

### Experimental design

The experimental design aimed to generate a group of pregnant and a group of non-inseminated cows 7 days after oestrus as previously described in Sponchiado *et al*.^9^. Thirty-six reproductively normal, cycling, non-lactating, multiparous Nelore (*Bos taurus indicus*) cows were used in this study. Animals were maintained under grazing conditions, supplemented with concentrate, chopped sugarcane, and minerals to fulfil their maintenance requirements. Animals had free access to water. Briefly, oestrous cycles were synchronized by i.m. administrations of 500 μg sodium cloprostenol (PGF_2α_ analogue; Ourofino Saúde Animal, Cravinhos, São Paulo, Brazil) and 2 mg oestradiol benzoate (Ourofino Saúde Animal), followed by insertion of an intravaginal P4-releasing device (1 g; Ourofino Saúde Animal). Eight days apart, the P4-releasing device was withdrawn, animals received an i.m. administration of 500 μg sodium cloprostenol and an Estrotect (Rockway, Inc. Spring Valley, WI, USA) heat detector patch. Between 48 and 84 h after P4-device removal, cows were checked for signs of oestrus activity twice a day. Only animals detected in oestrus were maintained in the experiment. On day zero (D0 = oestrus), cows were randomly assigned to the experimental groups: (i) Pregnant group (Preg; n = 16), cows were intracervically inseminated 12 h after oestrus, with frozen-thawed commercial semen of a proven fertility bull; or (ii) Control group (Con; n = 8), cows were sham-inseminated with semen extender. On D7, all animals were slaughtered.

Transrectal B-mode ultrasonography (7.5-MHz transducer) exams were conducted at 5 time points: at the time of P4-releasing device insertion and removal to measure follicles and to check the presence of a CL; on D0 and D1 to measure the size of the preovulatory follicle and to confirm ovulation, respectively. The side of the preovulatory follicle was recorded. On D7, the CL area and the first-wave largest follicle diameter were evaluated. As expected, pre-ovulatory follicle (on D0) and first-wave largest follicle (on D7) diameters did not differ between the two groups, nor the CL area and plasma P4 concentrations (on D7), as reported previously^9^.

### Uterine flushing and endometrial sample collection

**Figure 1:**
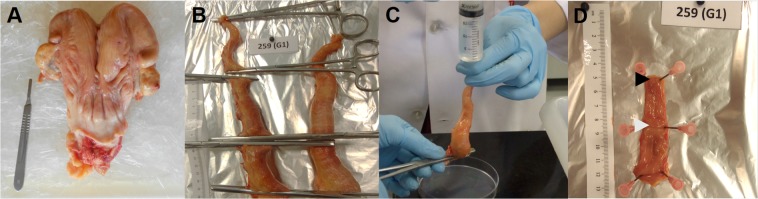
Diagram of sample collection procedure. (**A**) After slaughter, reproductive tracts were trimmed free of connective tissues; (**B**) The uterine horn ipsilateral to the corpus luteum was isolated. Starting from the utero-tubal junction (UTJ), locking tweezers were positioned every 8 cm to isolate the anterior, medial and posterior uterine thirds. (**C**) The ipsilateral anterior third was individually flushed by injecting 3 mL of PBS into the UTJ edge. (**D**) Intercaruncular endometrial samples were dissected from the UTJ (black arrow) and from the lengthwise intermediate region (white arrow) of the third, at the mesometrial side.

Animals were slaughtered by conventional captive bolt stunning followed by jugular exsanguination. The reproductive tracts were collected and transported on ice to the laboratory within 10 min. The uteri were trimmed free of adjacent tissues and processed for ULF and endometrial samples collection, as described previously^9^ and illustrated in Fig. 1. Briefly, the uterine horn ipsilateral to the ovary containing the CL was isolated. Starting from the utero-tubal junction (UTJ), locking tweezers were clamped every 8 cm to delimit the anterior, medial and posterior uterine thirds. Each portion was individually flushed by injecting ice-cold PBS into the cranial extremity and collecting the ULF at the caudal end in a petri dish. The anterior, medial and posterior thirds were flushed with 3, 5 and 6 mL of PBS, respectively. Inseminated cows were confirmed pregnants by visualization of an embryo in the ULF under a stereomicroscope. All embryos found (n = 10 out of 16) were in the ULF recovered exclusively from the ipsilateral anterior uterine third, and were at the expected developmental stage (compact morula or early blastocyst). Only inseminated cows from which an embryo was recovered were kept in the analyses. Uterine flushings were clarified by centrifugation at 1,000 × g for 10 min at 4 °C. The supernatant was gradually transferred into cryotubes, snap frozen and stored at −80 °C until analysis.

For endometrial samples collection, the ipsilateral anterior third was opened longitudinally along the mesometrial line. The endometrial surface was exposed and photographed for further surface area measurements. Intercaruncular endometrial fragments were dissected from the uterotubal junction (UTJ) and from the lengthwise intermediate region of the third, at the mesometrial side (Fig. 1). Tissue samples were transferred to cryotubes, snap frozen and stored at −80 °C until further processing.

### Targeted metabolomic measurements

We applied a targeted, quantitative metabolomics approach to analyse ULF samples (Con = 8; Preg = 10) from the anterior ipsilateral uterine third by using the AbsoluteIDQ p180, and Eicosanoid & Oxidized lipids mass spectrometry-based assays (Biocrates Life Sciences AG, Innsbruck, Austria). These validated assays enabled the simultaneous identification and quantification of up to 205 endogenous metabolites from seven analytic groups including 21 amino acids, 21 biogenic amines, 40 acylcarnitines, 90 glycerophospholipids, 15 sphingolipids, sum of hexoses, and 17 eicosanoids and oxidation products of polyunsaturated fatty acids. The assay procedure and metabolite nomenclature have been described in detail^17^. Briefly, the measurements were carried out in a 96-well plate with seven calibration standards and three quality control samples included. Amino acids and biogenic amines were analysed by UPLC (Waters ACQUITY UPLC, Waters Corporation, USA) system coupled with Xevo tandem quadrupole (TQ; Waters Corporation) and Xevo TQ-S mass spectrometers in positive mode. Acylcarnitines, glycerophospholipids, and sphingolipids were quantified by Waters tandem quadrupole mass spectrometers (Xevo TQ and Xevo TQ-S MS) by flow injection analysis (FIA) in positive mode, whereas hexoses were analysed using a subsequent acquisition in negative mode. Detection and quantification of the analytes was achieved using internal standards in multiple reaction monitoring (MRM) mode. Calculation of the metabolite concentrations analysed by FIA (acylcarnitines, glycerophospholipids, sphingolipids, and hexoses) was performed using Met*IDQ* software (Version 5– 4-8-DB100-Boron-2607, Biocrates Life Sciences AG). Analysis of peaks obtained by UPLC (amino acids and biogenic amines) was performed using TargetLynx Application Manager, and the results were imported into Met*IDQ* software for further processing.

Eicosanoids and related compounds were detected by Biocrates triple quadrupole MS-based platform in negative multiple-reaction monitoring detection mode as per a method reported previously^18^. Oxidized polyunsaturated fatty acids were extracted from ULF samples by methanolic protein precipitating process. Analysis was performed by HPLC MS/MS on a Sciex 5500 QTRAP™ (AP Sciex, Darmstadt, Germany) instrument. Metabolites were quantified by comparison to structurally similar molecules labelled with stable isotopes added to the samples in defined concentrations as internal standards in MRM mode.

### Metabolites panel

Throughout the article, amino acids are abbreviated based on their international notation. Acylcarnitines (Cx:y) are notated according to the fatty acid that is bound. Glycerophospholipids (sn) are classified according to the presence of ether (alkyl) or ester (acyl) residues attached to the glycerol moiety. The prefix ‘lyso’ denotes a single fatty acid or fatty alcohol bond on the sn-1 position of the glycerol moiety, as denoted by a single letter (acyl, a; alkyl, e). Two letters (diacyl, aa; acyl-alkyl, ae) means that the sn-1 and sn-2 positions on the glycerol moiety are each bound to a fatty acid or fatty alcohol residue. Sphingomyelins (SM) and hydroxysphingomyelins (SM-OH) are abbreviated based on the lipid chain composition (x:y). Biochemical name, abbreviation and PubChem CID of metabolites are listed in Supplemental Table S1 by class.

In addition to individual metabolite assessment, groups of metabolites were computed by sums or ratios of the amounts of analytes belonging to certain families or chemical structures to provide detailed insight into a wide range of functions. Details of ratios calculated and functional groups are provided in Supplemental Table S2.

### Total RNA isolation and transcript abundance analysis

Using a stainless-steel apparatus, frozen endometrial fragments (~40 mg) were mechanically minced. Immediately after, the macerate was homogenized with lysis buffer from PureLink® RNA mini kit (Ambion, Life Technologies, Carlsbad, California, USA), following manufacturer’s guidelines. The homogenate was passed ten times through a 21-ga needle accoupled to a 3 mL syringe to maximize lysis. Cell debris were removed by centrifugation at 12,000 g for 1 min at 4 °C. Subsequentially, the supernatant was loaded in RNeasy columns for further RNA isolation. Final RNA was eluted with 30 μL diethyl pyrocarbonate (DEPC)-treated water. Total RNA yield and purity were evaluated using NanoVue Plus Spectrophotometer (GE Healthcare, UK) by the absorbance at 260 nm and the 260/280 nm ratio, respectively.

Samples of RNA (400 ng) were subjected to treatment with DNase I Amplification Grade (Thermo Fisher Scientific) as per manufacturer’s instructions. Total RNA was reverse transcribed using Oligo(dT)12–18 Primers (Invitrogen) and dNTP Mix (Thermo Fisher Scientific). Samples were incubated at 65 °C for 5 min. First strand cDNA was synthesized adding the SuperScript IV Reverse Transcriptase (Thermo Fisher Scientific) to the RNA-primer mix, followed by incubation at 55 °C for 10 min and inactivation at 80 °C for 10 min. cDNA samples were stored at −20 °C.

The abundance of specific transcripts was determined by Real-Time PCR. Optimized primers were designed based on GenBank Ref-Seq (*Bos taurus*) mRNA sequences. Only primer pairs with an efficiency ranging from 90 to 110% were used. Primers assay efficiency was calculated based on the slope obtained from a standard curve (5-point serial dilution). Primers details are presented in Supplemental Table S3. Reactions were performed in triplicates in 96-well plates (Life Technologies), in a final volume of 20 μL. PCR amplification was carried out using the Step One Plus (Applied Biosystems, MA, USA) thermal cycler, using Power SYBR Green PCR Master Mix (Life Technologies). Cycling conditions were as follows. Initial denaturation was performed at 95 °C for 10 min, followed by 40 cycles of denaturation at 95 °C for 15 s and annealing reaction at 60 °C for 60 s. Melting curve analyses (from 60 to 95 °C) was performed to evaluate the amplification product. Negative controls (DEPC water replacing cDNA) were included in every run. Cycle thresholds (Cts) were determined using the LinReg PCR software as described by Ruijter *et al*.^19^. Target genes Ct values were normalized by the geometric mean of reference genes [actin beta (*ACTB*), peptidylprolyl isomerase A (*PPIA*), and glyceraldehyde-3-phosphate dehydrogenase (*GAPDH*)] transcript abundance values using the equation described by Pfaffl^20^.

### Data preparation: endometrial area measurements and normalization

To circumvent possible inaccuracies resulting from the flushing procedure due to different size of the uterine thirds between cows, we normalized the metabolite concentration values to the respective endometrial area. Endometrial area was measured with Image J 1.50i (National Institutes of Health, USA; http://rsb.info.nih.gov/ij/) software with the polygon selection. Prior to statistical analysis, metabolite concentration (nmol) values were adjusted by endometrial area (cm^2^) of the uterine third and are expressed as nmol/cm^2^.

### Bioinformatics and statistical analyses

For statistical analyses, only metabolites with more than 70% of their concentration values in the dynamic range were considered. Metabolic pathways and Multivariate data analyses were carried-out using the web-based metabolomic data processing tool MetaboAnalyst 4.0^21^. Metabolite concentrations were Pareto scaled and the method K-Nearest Neighbours was used to impute the missing values to generate the heatmap, sparse partial least squares-discriminate (sPLS-DA) and Metabolite Sets Enrichment analyses. Heatmap was set considering P-values between the two groups and the Ward’s methodology as clustering algorithm. For biological interpretation of the metabolite dataset, we mapped the quantified metabolites to the KEGG pathway database (Kyoto Encyclopedia of Genes and Genomes; www. genome.jp/kegg/). Metabolite Sets Enrichment Analysis was conducted on metabolite data mapped according to Human Metabolome Database (HMDB).

Univariate data analyses were carried-out using SAS software v. 9.3 (SAS Inst. Inc., Cary, NC, USA). Normal distribution and homogeneity of variances were ensured by Shapiro-Wilk and Welch’s test, respectively. Variables presenting heterogeneity of variances were transformed using natural logarithm. Concentrations and relative mRNA abundance were analysed for the main effect of group (Con *vs*. Preg) by two-tailed one-way ANOVA. To further validate the statistical significance, metabolite concentration P-values were subjected to False Discovery Rate (FDR) correction for multiple comparisons. The Q-value for FDR controlling procedure was set to 0.25^22^. Statistical significance was stated at P ≤ 0.05, and a probability of 0.05 < P ≤ 0.10 indicates a trend towards significance. Results are presented as means ± SEM.

## Results

### Metabolite profiling of ULF between pregnant *vs*. control cows

Targeted MS metabolomics was used to address the influence of one pre-hatching embryo on metabolite composition of ULF recovered from the ipsilateral anterior third on day 7 after oestrus. Of the 205 metabolites quantified, 167 were included in the analyses. Metabolites that were not detected in more than 30% of samples of both experimental groups were excluded from analyses.

#### Multivariate analyses: discriminant metabolomic signatures in the ULF from Control *vs.* Pregnant cows

**Figure 2:**
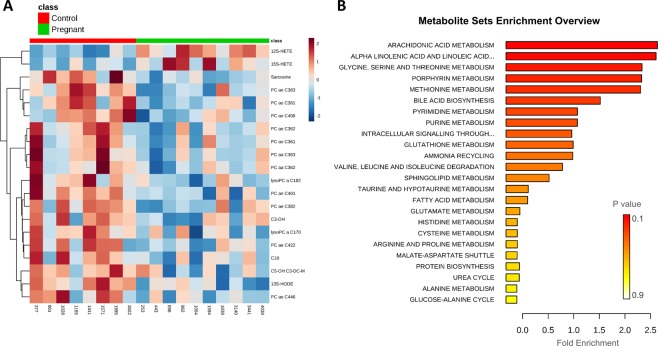
(**A**) Heatmap depicting the top 20 metabolites differently abundant between Pregnant and Control ULF samples based on *P*-values. (**B**) Quantitative Enrichment Analysis highlighted the metabolic pathways that were enriched in the Pregnant compared to the Control group, using the MetaboAnalyst 4.0 functional interpretation tools. The horizontal bars summarize the main metabolite sets identified in this analysis; the bars are coloured based on their P-values and the length is based on the -fold enrichment.

The heatmap shown in Fig. 2A revealed that: (i) the metabolomic profile of Preg cows is associated with an overall decrease of metabolite concentrations in the ULF compared to the Con group, and (ii) the clustering was affected mainly by compounds belonging to phospholipids, eicosanoids, acylcarnitines, amino acids and biogenic amines classes.

Metabolite Sets Enrichment Analysis was performed to determine, within the specific classes of metabolites measured, which biologically meaningful pathways were overrepresented. The dataset was mainly enriched for molecules involved in lipid and amino acid metabolism. The three top-score enrichment category were, in order of decreasing significance, (i) arachidonic acid metabolism; (ii) alpha linolenic acid and linoleic acid metabolism; and (iii) glycine, serine and threonine metabolism (Fig. 2B).

**Figure 3:**
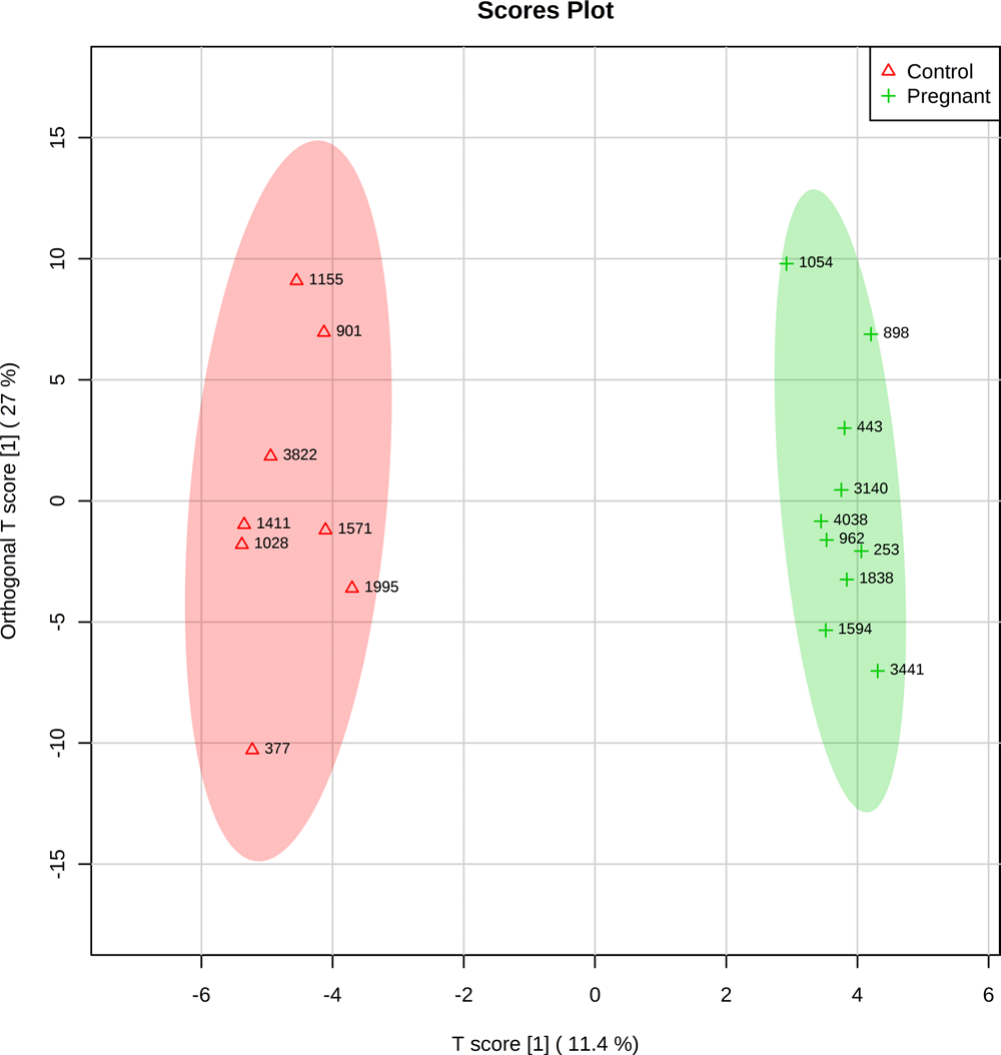
Ortho PLS-DA scatter plot depicting different ULF metabolomic profiles between Control and Pregnant cows on day 7 after oestrus. Each dot in the plot represents an animal according to the metabolite profile and groups are identified with ring ellipses corresponding to 95% confidence intervals.

Ortho PLS-DA performed on the metabolomics data revealed a clear discrimination between Preg and Con ULF profiles (Fig. 3), with the following parameters: R^2^X = 0.114, R^2^Y = 0.544, and Q^2^ = 0.348. All metabolites that passed quality control were included in this analysis. The top-seven most discriminant metabolites contributing to this model were: 12(S)-HETE, 15(S)-HETE, PC ae C42:5, DHA, LysoPC a C26:0, arachidonic acid (AA) and PGF_2a_.

#### Univariate analyses

**Figure 4:**
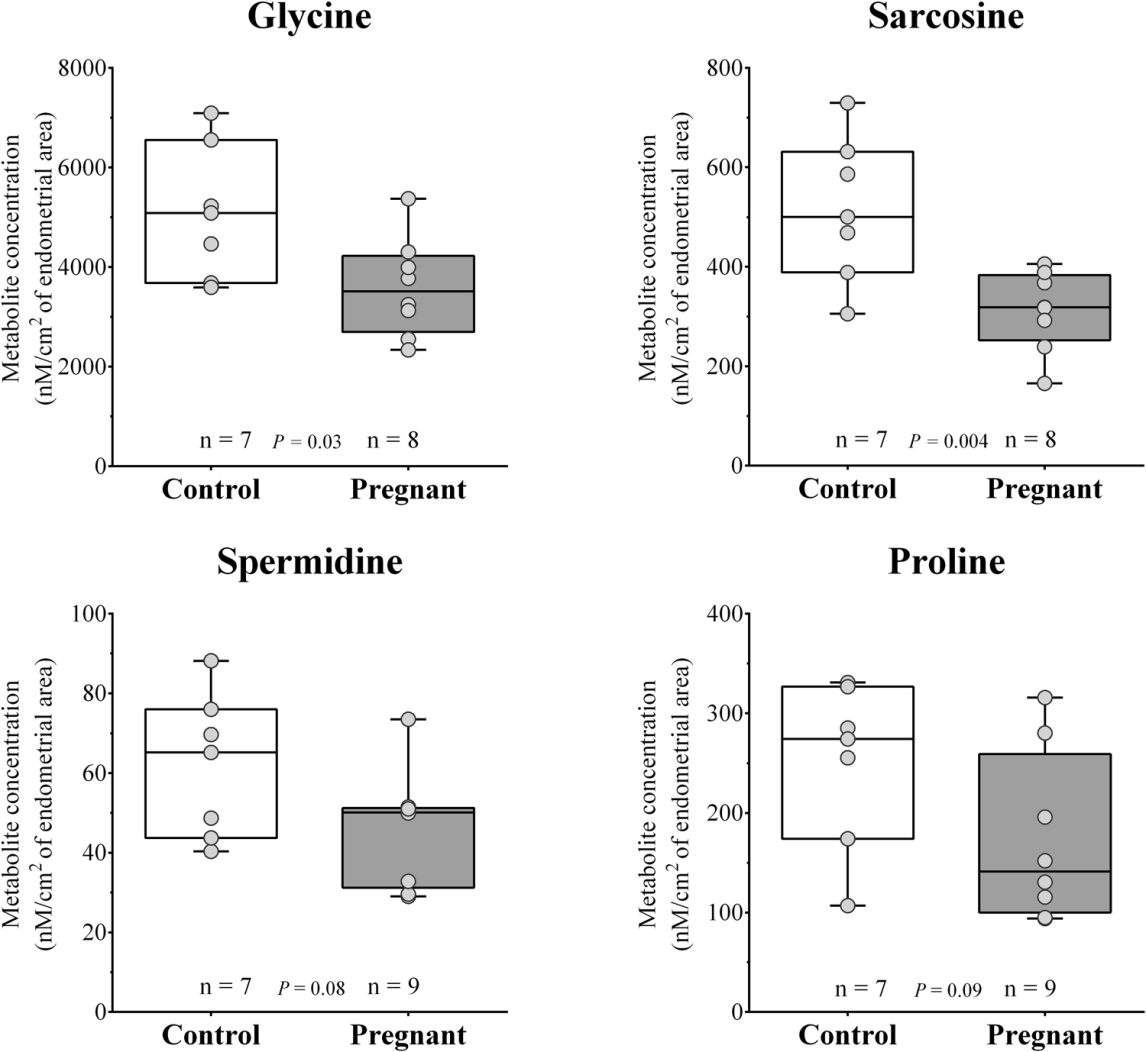
Box and whisker plots of amino acids and biogenic amines that show significant (*P* ≤ 0.05) or approaching (*P* ≤ 0.1) difference between Control and Pregnant uterine luminal fluids.

**Figure 5:**
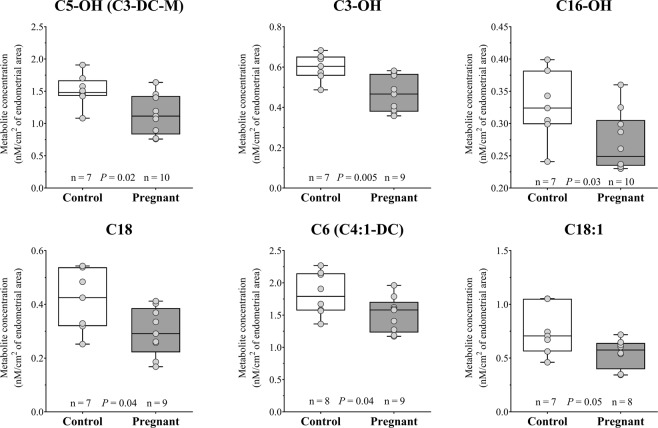
Box and whisker plots of acylcarnitines that show significant (*P* ≤ 0.05) or approaching (*P* ≤ 0.1) difference between Control and Pregnant uterine luminal fluids.

**Figure 6:**
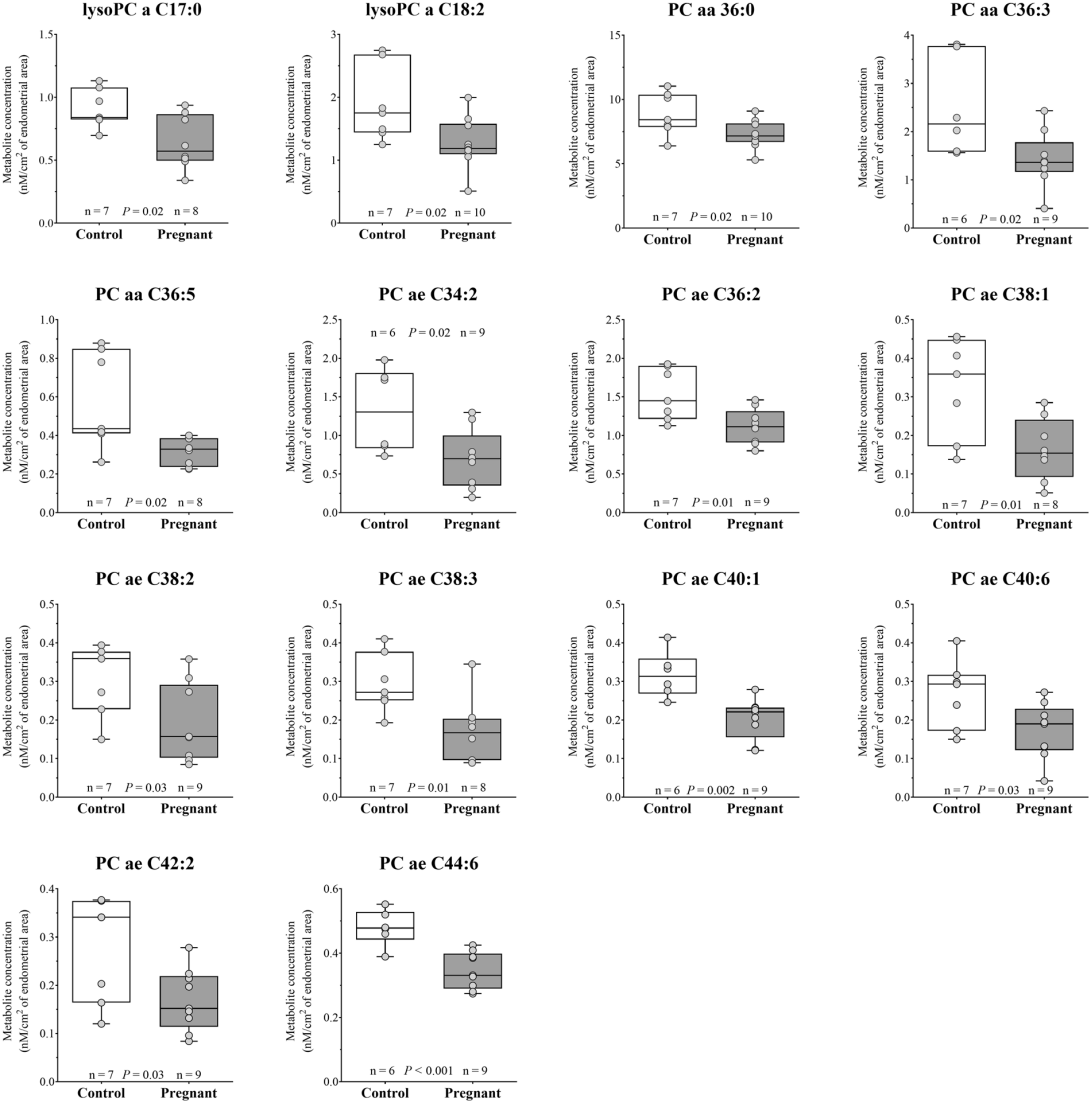
Box and whisker plots of phospholipids that show significant (*P* ≤ 0.05) difference between Control and Pregnant uterine luminal fluids.

**Figure 7:**
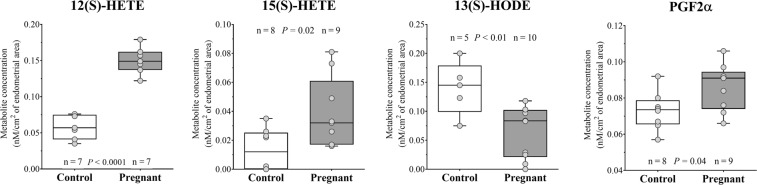
Box and whisker plots of eicosanoids and oxidation products of polyunsaturated fatty acids that show significant (*P* ≤ 0.05) difference between Control and Pregnant uterine luminal fluids.

Concentrations of single analytes and groups of analytes of a common biochemical classification were compared between the two experimental groups. Univariate analysis followed by FDR correction showed that, out of 167 metabolites that passed quality control, 22 (approximately 13%) showed significantly different concentrations (P ≤ 0.05; FDR corrected) between Con versus Preg cows. Remarkably, only two of these 22 metabolites were found in significantly increased concentration in ULF of Preg cows. Comparisons between the abundances of each metabolite according to pregnancy status are presented in Supplemental Tables S4 through S7 and Figs 4–7, according to biochemical classification.

#### Recoverable amounts of amino acids and biogenic amines in ULF

Concentrations of individual amino acids and biogenic amines according to group are presented in full in Supplemental Table S4. The non-essential amino acid glutamate was the most abundant in the uterine flushings (5323 nmol/cm^2^ of endometrial area) from both Con and Preg cows, followed by glycine (4293 nmol/cm^2^) and alanine (1182 nmol/cm^2^). Regarding the biogenic amines, taurine was the most prevalent (3705 nmol/cm^2^), followed by sarcosine (407 nmol/cm^2^) and putrescine (323 nmol/cm^2^). Total recoverable amounts of amino acids and biogenic amines (Table 1) were similar between ULF of Con and Preg cows. Of the amino acids and biogenic amines quantified, only glycine (0.7-fold; *P* ≤ 0.05) and sarcosine (0.6-fold; *P* ≤ 0.01), respectively, showed significantly decreased concentration in the ULF of Preg compared to Con cows, as shown in Fig. 4. Interestingly, glycine and sarcosine are both part of the glycine, serine and threonine metabolism pathway. Sums of small neutral and osmotic-stress protection amino acids were lower (*P* ≤ 0.05) 0.74 and 0.75-fold, respectively, in the Preg group compared to the controls.

**Table 1:** Sums and ratios of amino acids (AA) and biogenic amines (BA) concentrations in uterine luminal fluid from Control (Con) and Pregnant (Preg) cows.

Metabolite groups	Group		P value^a^	Log2 Fold-change^b^
	Con (n = 8)	Preg (n = 10)		
Total AA	19325.27 ± 1563.99	17447.25 ± 1895.85	0.36	−0.15
Non-essential AA	18861.09 ± 1469.75	17136.99 ± 1859.45	0.37	−0.14
Acidic AA	6766.67 ± 720.65	6154.43 ± 578.02	0.51	−0.14
**Small neutral AA**	10951.75 ± 903.36	8130.94 ± 655.04	0.02	−0.43
**Osmotic-stress protection AA**	11807.05 ± 940.68	8808.92 ± 710.92	0.02	−0.42
Glucogenic AA	6538.66 ± 630.91	5201.54 ± 661.67	0.17	−0.32
Glutathione precursors AA	11229.58 ± 997.77	9936.98 ± 996.13	0.39	−0.18
Total BA	5342.97 ± 442.49	4989.55 ± 675.82	0.70	−0.10
Spermidine/Putrescine^c^	0.18 ± 0.04	0.17 ± 0.02	0.83	−0.09
Spermine/Spermidine^d^	0.78 ± 0.16	0.57 ± 0.11	0.30	−0.43

Values are expressed as nmol/cm^2^ of endometrial area; mean ± SEM. Metabolite groups definitions are in Supplemental Table S2. Groups of metabolites in bold were different between Con and Preg.

^a^Statistical analyses were carried out by one-way ANOVA.

^b^Data are represented as fold-change of the metabolite concentration between Preg and Con groups.

^c^Ratio of Spermidine to Putrescine was calculated to access the activity of Spermidine synthase.

^d^Ratio of Spermine to Spermidine was calculated to access the activity of Spermine synthase.

#### Recoverable amounts of acylcarnitines in ULF

Concentrations of individual acylcarnitines according to group are presented in full in Supplemental Table S5. Short-chain acylcarnitines were the most abundant in ULF, followed by medium- and long-chain acylcarnitines (Table 2). Three acylcarnitines [Hydroxypropionylcarnitine (C3-OH), hydroxyisovalerylcarnitine (C5-OH), and hydroxyhexadecanolycarnitine (C16-OH)] of the 40 identified were found to be in significantly lower concentrations in the ULF from Preg cows (Fig. 5). Remarkably, all three metabolites that were different between the study groups were acylcarnitine esters derived from hydroxylated acids.

**Table 2:** Sums and ratios of carnitine and acylcarnitines (AC) concentrations in uterine luminal fluid from Control (Con) and Pregnant (Preg) cows.

Metabolite groups	Group		P value^a^	Log2 Fold-change^b^
	Con (n = 8)	Preg (n = 10)		
Total recoverable amounts of AC	239.56 ± 37.22	221.77 ± 25.88	0.69	−0.10
Total short-chain AC	75.86 ± 9.22	74.46 ± 9.22	0.92	−0.03
Total medium-chain AC	15.07 ± 1.23	13.43 ± 0.63	0.23	−0.17
Total long-chain AC	5.44 ± 0.52	4.80 ± 0.43	0.35	−0.18
Acylcarnitine/Free carnitine	0.68 ± 0.07	0.57 ± 0.04	0.22	−0.25
Total short-chain AC/Free carnitine	0.70 ± 0.07	0.64 ± 0.06	0.55	−0.14
CPT-I([C16 + C18]/C0)	0.046 ± 0.004	0.040 ± 0.004	0.32	−0.22
Total esters derived from DC/Total AC	0.032 ± 0.003	0.031 ± 0.003	0.84	−0.04
Esters derived from HO	4.69 ± 0.33	4.25 ± 0.29	0.33	−0.14
Esters derived from DC	6.98 ± 0.43	6.37 ± 0.32	0.25	−0.14

Acylcarnitines were categorized in esters derived from dicarboxylic acids (DC), esters derived from hydroxylated acids (OH), total short-chain acylcarnitines, total medium-chain acylcarnitines and total long-chain acylcarnitines. Values are expressed as nmol/cm^2^ of endometrial area; mean ± SEM. Metabolite groups definitions are in Supplemental Table S2.

^a^Statistical analyses were carried out by one-way ANOVA.

^b^Data are represented as fold-change of the metabolite concentration between Preg and Con groups.

#### Recoverable amounts of phosphatidylcholines and lysophosphatidylcholines in ULF

Concentrations of individual phospholipids according to group are presented in full in Supplemental Table S6. LysoPC a C14:0 was the most abundant phosphatidylcholine measured (243 nmol/cm^2^). Lysophosphatidylcholines represent 58% (Table 3) of the total phosphatidylcholines recovered in the ULFs. Total abundance of phosphatidylcholines (calculated by the sum of lyso-, diacyl- and acyl-alkyl-phosphatidylcholines; Table 3) was similar between ULF of Con and Preg cows. Total diacyl-phosphatidylcholines (P = 0.08) and polyunsaturated glycerophosphocholines (PUFA; P = 0.07) concentrations approached a significant reduction in the Preg group (Table 3). The ratio between PUFA and saturated (SFA) phosphatidylcholines showed a significant decrease in the Preg ULF (Table 3). This indicated that the activity of fatty acid desaturases of the endometrium might also be altered by pregnancy. There were 14 phosphatidylcholines identified with decreased concentration in the Preg group compared to its counterparts (Fig. 6), comprising 2 lyso-, 3 diacyl- and 9 acyl-alkyl-phosphatidylcholines. Interestingly, from the metabolites in different concentrations between groups, LysoPC a C18:2 and the diacyl-phosphatidylcholines (PC aa C36:0, PC aa 36:3, PC aa 36:5 and PC ae 36:2) are composed by one or two chains of stearic acid, a saturated fatty acid with an 18-carbon chain.

**Table 3:** Sums and ratios of Phospholipids concentrations in uterine luminal fluid from Control (Con) and Pregnant (Preg) cows.

Metabolite groups	Group		P value^a^	Log2 Fold-change^b^
	Con (n = 8)	Preg (n = 10)		
Total recoverable amounts of phospholipids	478.90 ± 37.61	429.32 ± 20.76	0.23	−0.15
Total recoverable amounts of LysoPC	297.98 ± 22.49	280.51 ± 13.05	0.49	−0.09
Total recoverable amounts of PC	182.30 ± 16.40	148.81 ± 9.81	0.08	−0.29
Total LysoPC/Total PC^c^	1.66 ± 0.13	1.92 ± 0.08	0.10	0.21
Total PC aa	108.42 ± 12.35	83.71 ± 7.22	0.08	−0.38
Total PC ae	73.88 ± 5.56	65.10 ± 3.13	0.16	−0.18
Total MUFA (PC)	41.44 ± 6.16	30.18 ± 3.57	0.11	−0.45
Total PUFA (PC)	84.33 ± 7.74	67.26 ± 4.87	0.07	−0.32
Total SFA (PC)	56.53 ± 4.45	51.37 ± 2.41	0.29	−0.14
MUFA (PC)/SFA (PC)^d^	0.74 ± 0.10	0.59 ± 0.06	0.20	−0.34
PUFA (PC)/MUFA (PC)^d^	2.19 ± 0.13	2.33 ± 0.12	0.45	0.08
**PUFA** (**PC**)**/SFA** (**PC**)^d^	1.42 ± 0.08	1.24 ± 0.03	0.04	−0.20

Phospholipids were grouped in Phosphatidylcholines (PC) and Lysophosphatidylcholines (LysoPC), diacyl- (PC aa) or acyl-alkyl- (PC ae) phosphatidylcholines, saturated (SFA), monounsaturated (MUFA) and polyunsaturated (PUFA) glycerophosphocholines. Values are expressed as nmol/cm^2^ of endometrial area; mean ± SEM. Metabolite groups definitions are in Supplemental Table S2.

Groups of metabolites in bold were different between Con and Preg group.

^a^Statistical analyses were carried out by one-way ANOVA.

^b^Data are represented as fold-change of the metabolite concentration between Preg and Con groups.

^c^Ratio of LysoPC to PC is an indicator of phospholipase activity.

^d^Ratios of MUFA to SFA, PUFA to MUFA, and PUFA to SFA were measures of the activity of fatty acid desaturases.

#### Recoverable amounts of sphingolipids in ULF

Concentrations of individual sphingolipids according to group are presented in full in Supplemental Table S7. We detected six species of sphingomyelins (C16:0, C16:1, C18:0, C18:1, C24:0 and C24:1) and four species of hydroxysphingomyelins (C16:1, C22:1, C22:2 and C24:1) in ULF obtained from both groups. Sphingomyelin C16:0 was the most abundant in the uterine flushings (19.84 nmol/cm^2^ of endometrial area). There was no difference in the concentration of any sphingolipid between Con and Preg cows. However, the total recoverable amount of hydroxysphingomyelins tended to be lower (0.67-fold; P ≤ 0.1; Table 4) in the Preg ULF samples.

**Table 4:** Sums and ratios of Sphingolipids concentrations in uterine luminal fluid from Control (Con) and Pregnant (Preg) cows.

Metabolite groups	Group		P value^a^	Log2 Fold-change^b^
	Con (n = 8)	Preg (n = 10)		
Total SM	36.87 ± 6.50	28.84 ± 4.49	0.31	−0.36
**Total SM-OH**	4.76 ± 0.85	3.18 ± 0.43	0.09	−0.58
Ratio SM/SM-OH	7.85 ± 0.39	9.02 ± 0.51	0.13	0.20
Total unsaturated SM	5.40 ± 0.96	4.31 ± 0.68	0.36	−0.32
Total saturated SM	31.47 ± 5.55	24.53 ± 3.85	0.31	−0.36

Metabolites were grouped in sphingomyelins (SM) and hydroxysphingomyelins (SM-OH) and according to the unsaturation. Values are expressed as nmol/cm^2^ of endometrial area; mean ± SEM. Metabolite groups definitions are in Supplemental Table S2.

Group of metabolites in bold tended to be different between Con and Preg group.

^a^Statistical analyses were carried out by one-way ANOVA.

^b^Data are represented as fold-change of the metabolite concentration between Preg and Con groups.

#### Recoverable amounts of hexoses in ULF

In this study, hexoses were the most abundant metabolite class identified in the ULF (334,474.62 nmol/cm^2^ of endometrial area). There was no difference in hexoses concentration between Con and Preg ULF samples (Supplemental Table S8).

#### Recoverable amounts of eicosanoids and oxidation products of polyunsaturated fatty acids in ULF

Eicosanoid and oxidation products of polyunsaturated fatty acids profiling in ULF from Con and Preg cows is presented in Tables 5 and 6. Arachidonic acid, an omega-6 fatty acid, was the most abundant polyunsaturated fatty acid in uterine flushings from both Con and Preg groups, followed by Docosahexaenoic acid, an omega-3 fatty acid. Multiple cyclooxygenase, lipoxygenase, and cytochrome P450 metabolite products were identified. Regarding cyclooxygenase metabolite products, only PGF_2α_ and PGI_2_ (inferred from measurement of 6-keto-Prostaglandin F1alpha) were detected in the ULF samples, while PGE_2_, PGD_2_, PGEM and TXB_2_ were below the limit of detection. Univariate analysis revealed a main effect of group between Con and Preg ULF for lipoxygenase metabolite products, 12(S)-HETE, 15(S)-HETE and 13(S)-HODE, wherein 12(S)-HETE and 15(S)-HETE were 2.54 and 2.84-folds greater in the Preg ULF, respectively (Fig. 7). However, 13(S)-HODE was 0.46-fold in lower concentration in ULF from the Preg group. Regarding cyclooxygenase metabolite products, PGF_2α_ concentration tended to be higher (1.18-fold) in the Preg group.

**Table 5:** Eicosanoids and oxidation products of polyunsaturated fatty acids concentration in uterine luminal fluid from Control (Con) and Pregnant (Preg) cows.

Metabolite	Group		P value	FDR significance^a^	Log2 Fold-change^b^
	Con (n = 8)	Preg (n = 10)			
Arachidonic acid	12.95 ± 1.81	18.27 ± 0.90	0.18	n.s.	0.50
Docosahexaenoic acid (DHA)	2.58 ± 0.25	3.47 ± 0.11	0.06	n.s.	0.43
**13**(**S**)**-HODE**^c^	0.14 ± 0.02	0.06 ± 0.004	0.009	*	−1.12
**12**(**S**)**-HETE**^c^	0.06 ± 0.006	0.15 ± 0.003	0.0001	**	1.34
**15**(**S**)**-HETE**^c^	0.013 ± 0.005	0.038 ± 0.003	0.02	*	1.51
6-keto-Prostaglandin F1alpha (PGI_2_)^d^	0.08 ± 0.01	0.09 ± 0.002	0.74	n.s.	0.06
Prostaglandin F2alpha (PGF_2α_)^d^	0.07 ± 0.004	0.09 ± 0.001	0.04	n.s.	0.24

Values are expressed as nmol/cm^2^ of endometrial area; mean ± SEM. Biochemical name, abbreviation and PubChem CID of metabolites are listed in Supplemental Table S1 by class.

Metabolites in bold were different between Con and Preg group by ANOVA followed by FDR correction.

^a^Statistical analyses were carried out by one-way ANOVA followed by FDR correction for multiple comparisons. Magnitude of effect is indicated by: **P ≤ 0.01; *P ≤ 0.05; n.s. *P* > 0.05.

^b^Data are represented as fold-change of the metabolite concentration between Preg and Con groups.

^c^Lipoxygenase metabolite products.

^d^Cyclooxygenase metabolite products.

**Table 6:** Sums of eicosanoids and oxidation products of polyunsaturated fatty acids concentrations in uterine luminal fluid from Control (Con) and Pregnant (Preg) cows.

Metabolite groups	Group		P value^a^	Log2 Fold-change^b^
	Con (n = 8)	Preg (n = 10)		
COX pathway	0.152 ± 0.009	0.167 ± 0.007	0.20	0.14
LOX pathway	0.224 ± 0.041	0.225 ± 0.031	0.98	0.00

Metabolites were grouped according to it derivation from the cycloxygenase (COX) and lipoxygenase (LOX) pathways. Values are expressed as nmol/cm^2^ of endometrial area; mean ± SEM. Metabolite groups definitions are in Supplemental Table S2.

^a^Statistical analyses were carried out by one-way ANOVA.

^b^Data are represented as fold-change of the metabolite concentration between Preg and Con groups.

### Transcript abundance on endometrial samples

**Figure 8:**
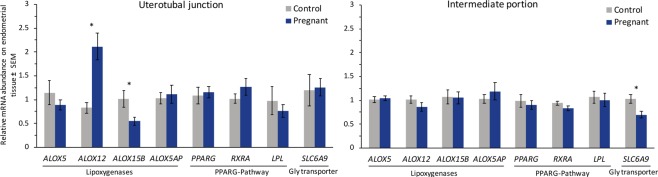
Relative mRNA abundance of Lipoxygenases, PPARG-pathway associated genes and Glycine Transporter in Control and Pregnant endometrial samples dissected from the uterotubal junction and the lengthwise intermediate portion of the anterior third of the ipsilateral uterine horn. Data are shown as arbitrary units; mean ± SEM.

Because of the significantly greater abundance of lipoxygenase-related metabolites in ULF from Pregnant cows, we measured the abundance of transcripts coding for Lipoxygenase enzymes (*ALOX5*, *ALOX5AP*, *ALOX15B* and *ALOX12*), and lipoxygenase metabolite targets (*PPARG*, *RXRA* and *LPL*) in endometrial samples by Real Time PCR. Additionally, because of the lower abundance of glycine in the ULF from Pregnant cows, we measured the abundance of mRNA for a Glycine Transporter (*SLC6A9*). Transcripts for *ALOX12* and *ALOX15B* were, respectively, 2.56-folds up- and 0.54-fold down-regulated in the UTJ from Pregnant animals (Fig. 8). All the Lipoxygenases addressed in this study were similar between groups in the endometrium collected from the intermediate region of the anterior third. However, a decreased abundance of *SLC6A9* (a Glycine transporter; 0.76-fold) transcripts was found in the Preg endometrial tissue in the intermediate region, suggesting an embryo-modulated, endometrial response that was consistent with the lower Glycine concentration in the ULF. Transcripts for *PPARG* and *RXRA* were detected in endometrial tissue, as expected, but there was no effect of group on mRNA abundance of *PPARG* and *RXRA* in neither the UTJ or the intermediate anterior third endometrial samples. Similarly, mRNA abundance of *LPL*, a target-gene of PPARG-RXRA complex activation was not affected by group.

## Discussion

Accumulating evidence supports the idea that the early bovine embryo is more than a passive passenger through the maternal reproductive tract. Embryo-induced effects, at transcriptional level, on oviductal^13^, endometrial^9–12^, luteal^14^ and immune^15,16^ cells have been reported. In the present study, we further advanced the understanding of the embryo effects in early pregnancy. We demonstrated for the first time that the pre-hatching embryo changes the uterine microenvironment as early as day 7 after oestrus *in vivo*. In the present study, we had the unique opportunity to collect ULF from the anterior third of the uterine horn. Consequently, metabolite concentrations measured represent the local concentrations. This is critical because due to the cranial-most localization of the embryo at day 7, it is possible that a greater magnitude of effects of the embryo were manifest in its closest proximity. Such modulation included changes in concentrations of lipoxygenase-derived metabolites, amino acids, biogenic amines, acylcarnitines and phospholipids. The changed composition of the ULF could be due to secretion or depletion of specific molecules, executed by either the embryo or the endometrium, but initiated by signals coming from the embryo. Another major contribution of our study is the expanded inventory and absolute quantification of naturally occurring compounds on ULF. This is important, because most published metabolomics-based investigations are either restricted to one class of analytes and only relative abundances are reported.

Multivariate analyses showed a clear separation between animals pertaining to the two experimental groups. This indicates that the pre-hatching embryo changes the global ULF metabolome profile *in vivo*. *Eicosanoids and oxidation products of polyunsaturated fatty acids* was the main biochemical class contributing to the discriminant model. Univariate analyses revealed 22 metabolites displaying different concentrations between Preg and Con ULF samples after FDR correction. If all comparisons showing P ≤ 0.05 were considered regardless of FDR correction, we would have detected 33 analytes with different abundances between Preg and controls. Out of the 22 metabolites showing different concentrations among the two experimental groups, 20 were in lower concentration in the ULF recovered from Pregnant animals. *In vivo*, the ULF composition is modulated by both the endometrial and embryonic units, as well as their molecular interactions. Lower concentrations of metabolites in ULF samples could be attributed to at least four possibilities: (1) a hypermetabolic state of the endometrium, that resulted in increased consumption of substrates by the endometrial tissue with a consequent reduced transport towards the lumen; (2) an overall down-regulation of transport activity in the endometrial epithelia; (3) an increased resorption of metabolites from the uterine lumen towards the lining endometrial epithelium; (4) intake of compounds present in the uterine fluid by the embryo; or combinations of the above. Validation of such hypothetical mechanisms requires further investigation. Most pregnancy-induced differences were found in eicosanoids, amino acids, biogenic amines, acylcarnitines and phospholipid classes.

The pre-hatching embryo modulates the eicosanoid metabolism in the uterine lumen. Pregnant animals presented greater concentrations of 12(S)-HETE and 15(S)-HETE, but decreased amounts of 13(S)-HODE. These metabolites are products of the oxidative metabolism of arachidonic and linoleic acids generated by the lipoxygenases (LOX) enzymes. Biological activities of lipoxygenase-products include neovascularization and vasodilatation, regulation of inflammatory response and immune function, control of oxidative stress and lipid metabolism^23^. Synthesis of lipoxygenase products by uterine tissues and their role on early embryo development have not been studied extensively. In mice, it has been shown that complete blockade of uterine 12/15-LOX activity by a specific inhibitor reduced uterine levels of arachidonic acid metabolites and impaired implantation by 80% compared to untreated controls^24^. In cattle, Ribeiro *et al*.^25^, have shown that the ULF recovered from pregnant dairy cows had increased amounts of 15(S)-HETE compared to their non-pregnant counterparts on day 15. In the present study, we showed for the first time that presence of the pre-implantation embryo is capable of modulating the abundance of lipoxygenase-derived metabolites in the uterine lumen. This entices us to speculate that such metabolites may induce changes in function of target tissues, such as the embryo and the endometrium.

We next explored whether changes in the ULF abundance of lipoxygenase-derived metabolites were associated with changes in LOX transcript abundance in the endometrium. Endometrial abundances of *ALOX12* and *ALOX15B* transcripts were respectively up- and downregulated in the UTJ of Preg vs. Con animals. In our previous study, we demonstrated that the main effects of the D7 embryo on the endometrial transcriptome were mainly in the UTJ^9^. Our findings are consistent with those from a recent study comparing the transcriptome response of the endometrium to pregnancy between high fertile- and subfertile-classified heifers^26^. In that study, the pregnant endometrium displayed an up-regulated expression of *ALOX5AP* and *ALOX12*, and a down-regulation in the expression of *ALOX15* and *ALOX15B* compared to non-pregnant endometrium on day 17^26^. Moreover, an upregulation of *ALOX5AP* was detected on endometrial tissue of pregnant heifers on day 16 of pregnancy compared to their non-pregnant counterparts^27^. Taken together, these studies prompt to the idea that embryo/conceptus-derived signals are capable to modulate the endometrial expression of lipoxygenases and, ultimately, affect the lipoxygenase-derived products concentration in the ULF. Studies are needed to elucidate the mechanisms of regulation and potential roles of Lipoxygenase-derived metabolites, as well as their associated signalling systems, in the endometrium and in the embryo during early pregnancy.

We subsequently investigated whether the changed abundance of lipoxygenase-derived metabolites influenced PPARγ signalling in the endometrium. Both 12(S)-HETE and 15(S)-HETE have been shown to be endogenous ligands/activators of PPARγ transcription factor *in vivo*^24^ and *in vitro*^28^. In the report by Li *et al*.^24^, the impaired implantation in mice pre-treated with 12/15-LOX inhibitor was restored by administration of rosiglitazone, a PPARγ agonist. In the present paper, we initially confirmed the expression of *PPARG* and *RXRA* mRNA in the endometrium on day 7. Next, we examined the endometrial abundance of *LPL* mRNA, a PPARγ target-gene, but failed to detect a difference in the abundance of that transcript between Preg and Con samples. This suggests a lack of regulation of this system by the embryo at this stage of pregnancy.

The pre-hatching embryo decreases abundance of specific amino acids and biogenic amines in the uterine lumen. Of the amino acids and biogenic amines quantified, glycine and sarcosine were present in significantly reduced concentrations in the ULF of Preg cows. We also verified that the sums of small neutral, and osmotic-stress protection amino acids were lower in Preg compared to the Con group. This may seem at odds with evidence in the literature that amino acids are increased in the uterine lumen in cattle^29,30^ and sheep^31^ during the peri-implantation period which is mediated via upregulation of amino acid transporters in the endometrium. However, compared with previous work, the present samples were collected approximately one week earlier in gestation. Interestingly, glycine and sarcosine are both part of the glycine, serine and threonine metabolism pathway. Sarcosine is an intermediate and byproduct in glycine synthesis and degradation. Glycine is inter-convertible to Serine and Alanine and is furthermore necessary for protein and DNA synthesis (i.e., for cell proliferation), also acts as an intracellular regulator and as an organic osmolyte^32^. Brison *et al*.^33^ found that Glycine is less abundant in the culture medium of human embryos that resulted in successful IVF pregnancies. Regarding the mediated transport of Glycine to the uterine lumen, Hugentobler *et al*.^34^ have shown that ULF concentration of glycine on day 6 of the oestrous cycle was greater and not correlated to its concentration in blood plasma. This pattern indicates that glycine is actively transported by the endometrial epithelia towards the uterine lumen. In the present study, we detected a downregulation of *SLC6A9* (Glycine transporter, also known as *GLYT1*) transcript in the Preg endometrial tissue, suggesting an endometrial origin of regulation that was consistent with the lower Glycine concentration in the ULF. Mechanisms by which the embryo regulates this process are currently unknown.

Presence of a day-7 embryo decreases the concentration of acylcarnitines and phospholipids in the ULF. Acylcarnitines are key molecules enrolled in fatty acids transport and energy metabolism. Notably, three acylcarnitines (C3-OH, C5-OH and C16-OH), that were found in different concentrations between groups, are esters derived from hydroxylated acids. Hydroxylated acylcarnitine status is an important indicator of lipid metabolism by the fatty acid omega-oxidation pathway and may represent an important biomarker of fatty acid metabolism^35^. Phosphatidylcholines are important structural components of plasma lipoproteins and cell membranes, and have important roles in the regulation of cell function and signalling^36^. The ratio between PUFA and SFA showed to be decreased in Preg ULF samples. This finding indicates that the activity of fatty acid desaturases between Con and Preg endometria may be altered. Decreased levels of phospholipids in biological fluids might be attributable to enhanced cell membrane synthesis in the lining cellular compartments^37^. Differences in lipid profile in the endometrium at late diestrus between pregnant and non-pregnant ewes^38^, and between gravid and non-gravid horns of pregnant cows^39^ have been reported before. In our study, we showed that both LysoPC a C17:0 and LysoPC a C20:3 fatty acids were significantly lower in abundance in ULF recovered from Preg compared to Con cows. It is possible that these lipids were partially retained by the lining endometrium. Meier *et al*.^40^ have shown that endometrial tissue from pregnant cows displayed greater concentrations of C17:0 and C20:3 fatty acids compared to their cyclic counterparts on day 17 after oestrus. Interestingly, the C20:3 fatty acid acts as precursors for prostaglandin synthesis^41^. Additionally, the total recoverable amount of hydroxysphingomyelins tended to be greater in the Con ULF samples. Stimulated synthesis of sphingolipids is related to a pro-apoptotic status of the endometrium in women^42^. At the present time, we can only speculate on the functional relevance and regulation of changes in the abundance of specific lipids. Much more research is needed on the topic of lipid biology of pregnancy.

**Figure 9:**
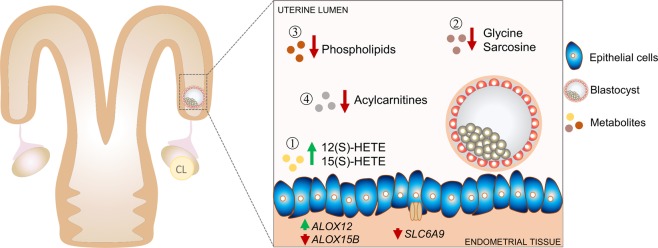
Summary and integration of the main results. Metabolomic measurements reveal an overall decrease in substrate concentration in uterine luminal fluid recovered from pregnant (Preg) compared to control cyclic (Con) cows on day 7 post oestrus. (1) Concentrations of two metabolites 12(S)-HETE and 15(S)-HETE, associated to the lipoxygenases pathway, were significantly greater in the Preg group. Transcripts for lipoxygenases were up- (*ALOX12*) and downregulated (*ALOX15B*) in the uterotubal junction of Preg animals, suggesting an endometrial origin of regulation of Lipoxygenases-derived metabolites in ULF. (2) Glycine and sarcosine were significantly lower in abundance in ULF recovered from Preg compared to Con cows. A downregulation of *SLC6A9* (a Glycine transporter) transcripts was found in the Preg endometrial tissue, that was consistent with the lower Glycine concentration in ULF. Exposure to a day 7 embryo modulates the concentration of (3) phospholipids and (4) acylcarnitines concentration in ULF. We propose that metabolite composition of the ULF changes in response to a pre-hatching embryo *in vivo*.

In conclusion, we produced evidence to sustain the view that the bovine embryo modulates the biochemical composition of the uterine microenvironment as early as day 7 *in vivo*. Such modulation seems to be local and includes changes in concentrations of lipoxygenase-derived metabolites, amino acids, biogenic amines, acylcarnitines and phospholipids (Fig. 9). Although the endometrial or embryonic origin of the modulated biochemical processes can only be speculated, it is clear that regulation is complex and interactive. Intensity and extent of embryo signalling capacity is expected to increase dynamically throughout the window of pre-implantation development, in order to cover its changing needs. Altogether, the data of our *in vivo* model highlighted key pathways involved in early embryo-induced changes in the luminal uterine metabolome. Of particular interest, the products of the lipoxygenase-pathway seem to play an important role in early pregnancy. This novel finding warrants further investigation.

## Supplementary information

Supplementary information
