# Supplementary material for: The pre-hatching bovine embryo transforms the uterine luminal metabolite composition in vivo
Source: Sci Rep. 2019 Jun 7;9:8354. doi: 10.1038/s41598-019-44590-9 (PMC6555789; doi:10.1038/s41598-019-44590-9)
Supplement: Supplementary file 1 — Supplementary information [file 41598_2019_44590_MOESM1_ESM.pdf]

The pre-hatching bovine embryo transforms the uterine luminal metabolite composition in vivo

Mariana Sponchiado, Angela M. Gonella-Diaza, Cecília C. Rocha, Edson G. Lo Turco, Guilherme Pugliesi, Jo L. M. R. Leroy, Mario Binelli

Supplementary Table S1. Biochemical name, abbreviation and PubChem Compound ID of each metabolite quantified in uterine luminal fluid samples recovered from Control and Pregnant cows *post mortem*.

Metabolite	Abbreviation	PubChem CID
Acylcarnitines		
Carnitine (free)	C0	2724480
Decanoylcarnitine [= Caprylcarnitine]	C10	10245190
Decenoylcarnitine	C10:1	53481651
Decadienoylcarnitine	C10:2	53481669
Dodecanoylcarnitine [= Laurylcarnitine]	C12	168381
Dodecanedioylcarnitine	C12-DC	53481673
Dodecenoylcarnitine	C12:1	53481671
Tetradecanoylcarnitine [= Myristylcarnitine]	C14	53477791
Tetradecenoylcarnitine [= Myristoleylcarnitine]	C14:1	22833575
Hydroxytetradecenoylcarnitine [= Hydroxymyristoleylcarnitine]	C14:1-OH	53481679
Tetradecadienoylcarnitine	C14:2	71464539
Hydroxytetradecadienoylcarnitine	C14:2-OH	71464482
Hexadecanoylcarnitine [= Palmitoylcarnitine]	C16	11953816
Hydroxyhexadecanolycarnitine [= Hydroxypalmitoylcarnitine]	C16-OH	24779579
Hexadecenoylcarnitine [= Palmitoleylcarnitine]	C16:1	53477817
Hydroxyhexadecenoylcarnitine [= Hydroxypalmitoleylcarnitine]	C16:1-OH	53481779
Hexadecadienoylcarnitine	C16:2	53481687
Hydroxyhexadecadienoylcarnitine	C16:2-OH	53481689
Octadecanoylcarnitine [= Stearylcarnitine]	C18	6426855
Octadecenoylcarnitine [= Oleylcarnitine]	C18:1	53477837
Hydroxyoctadecenoylcarnitine [= Hydroxyoleylcarnitine]	C18:1-OH	53481697
Octadecadienoylcarnitine [= Linoleylcarnitine]	C18:2	53477834
Acetylcarnitine	C2	1
Propionylcarnitine	C3	107738
Hydroxybutyrylcarnitine (Malonylcarnitine)	C3-DC (C4-OH)	22833583
Hydroxypropionylcarnitine	C3-OH	53481613
Propenoylcarnitine	C3:1	53481611
Butyrylcarnitine / Isobutyrylcarnitine	C4	439829
Butenoylcarnitine	C4:1	4151505
Isovalerylcarnitine / 2-Methylbutyrylcarnitine / Valerylcarnitine	C5	6426851
Glutarylcarnitine (Hydroxyhexanoylcarnitine [= Hydroxycaproylcarnitine])	C5-DC (C6-OH)	53481622
Methylglutarylcarnitine	C5-M-DC	128145
Hydroxyisovalerylcarnitine / Hydroxy-2-methylbutyryl / Hydroxyvalerylcarnitine (Methylmalonylcarnitine)	C5-OH (C3-DC-M)	53481628
Tiglylcarnitine / 3-Methyl-crotonylcarnitine	C5:1	22833596
Glutaconylcarnitine / Mesaconylcarnitine	C5:1-DC	53481620

Hexanoylcarnitine [= Caproylcarnitine] (Fumarylcarnitine)	C6 (C4:1-DC)	3246938
Hexenoylcarnitine	C6:1	53481638
Pimelylcarnitine	C7-DC	53481675
Octanoylcarnitine [= Caprylylcarnitine]	C8	11953814
Nonanoylcarnitine [= Pelargonylcarnitine]	C9	53481660
Amino Acids & Biogenic Amines		
Alanine	Ala	5950
Arginine	Arg	6322
Asparagine	Asn	6267
Aspartate	Asp	5960
Carnosine	Carnosine	439224
Citrulline	Cit	9750
Creatinine	Creatinine	588
Dopamin	Dopamine	681
Glutamine	Gln	5961
Glutamate	Glu	33032
Glycine	Gly	750
Histamine	Histamine	774
Leucine	Leu	6106
Proline	Pro	145742
Putrescine	Putrescine	1045
Sarcosine	Sarcosine	1088
Symmetric dimethylarginine	SDMA	169148
Serine	Ser	5951
Spermidine	Spermidine	1102
Spermine	Spermine	1103
trans-4-Hydroxyproline	t4-OH-Pro	5810
Taurine	Taurine	1123
Threonine	Thr	6288
Eicosanoids & Oxidation products of polyunsaturated fatty acids		
12(S)-hydroxy-5Z,8Z,10E,14Z-eicosatetraenoic acid	12S-HETE	5283155
13(S)-hydroxy-9Z,11E-octadecadienoic acid	13S-HODE	6443013
15(S)-hydroxy-5Z,8Z,11Z,13E-eicosatetraenoic acid	15S-HETE	5280724
6-keto-Prostaglandin F1alpha	6-keto-PGF1a	5280888
Arachidonic acid	AA	444899
Docosahexaenoic acid	DHA	445580
Prostaglandin F2alpha	PGF2a	5282415
Hexoses		
Hexoses	H1	.
Phosphatidylcholines		
Lysophosphatidylcholine with acyl residue C14:0	lysoPC a C14:0	460604
Lysophosphatidylcholine with acyl residue C16:0	lysoPC a C16:0	10917802
Lysophosphatidylcholine with acyl residue C16:1	lysoPC a C16:1	24779461
Lysophosphatidylcholine with acyl residue C17:0	lysoPC a C17:0	24779463

Lysophosphatidylcholine with acyl residue C18:0	lysoPC a C18:0	2733532
Lysophosphatidylcholine with acyl residue C18:1	lysoPC a C18:1	53480465
Lysophosphatidylcholine with acyl residue C18:2	lysoPC a C18:2	11005824
Lysophosphatidylcholine with acyl residue C20:3	lysoPC a C20:3	52924055
Lysophosphatidylcholine with acyl residue C20:4	lysoPC a C20:4	53480469
Lysophosphatidylcholine with acyl residue C24:0	lysoPC a C24:0	24779481
Lysophosphatidylcholine with acyl residue C26:0	lysoPC a C26:0	44340994
Lysophosphatidylcholine with acyl residue C26:1	lysoPC a C26:1	52925041
Lysophosphatidylcholine with acyl residue C28:0	lysoPC a C28:0	52924960
Lysophosphatidylcholine with acyl residue C28:1	lysoPC a C28:1	52923870
Phosphatidylcholine with diacyl residue sum C24:0	PC aa C24:0	6452499
Phosphatidylcholine with diacyl residue sum C26:0	PC aa C26:0	52924957
Phosphatidylcholine with diacyl residue sum C28:1	PC aa C28:1	52922210
Phosphatidylcholine with diacyl residue sum C30:0	PC aa C30:0	24778679
Phosphatidylcholine with diacyl residue sum C32:0	PC aa C32:0	131150
Phosphatidylcholine with diacyl residue sum C32:1	PC aa C32:1	24778618
Phosphatidylcholine with diacyl residue sum C32:2	PC aa C32:2	52922262
Phosphatidylcholine with diacyl residue sum C32:3	PC aa C32:3	52922763
Phosphatidylcholine with diacyl residue sum C34:1	PC aa C34:1	53478717
Phosphatidylcholine with diacyl residue sum C34:2	PC aa C34:2	53478719
Phosphatidylcholine with diacyl residue sum C34:3	PC aa C34:3	52922280
Phosphatidylcholine with diacyl residue sum C34:4	PC aa C34:4	52922891
Phosphatidylcholine with diacyl residue sum C36:0	PC aa C36:0	94190
Phosphatidylcholine with diacyl residue sum C36:1	PC aa C36:1	52922290
Phosphatidylcholine with diacyl residue sum C36:2	PC aa C36:2	15378085
Phosphatidylcholine with diacyl residue sum C36:3	PC aa C36:3	52922727
Phosphatidylcholine with diacyl residue sum C36:4	PC aa C36:4	53478831
Phosphatidylcholine with diacyl residue sum C36:5	PC aa C36:5	52922687
Phosphatidylcholine with diacyl residue sum C36:6	PC aa C36:6	53478633
Phosphatidylcholine with diacyl residue sum C38:0	PC aa C38:0	52923443
Phosphatidylcholine with diacyl residue sum C38:1	PC aa C38:1	53478731
Phosphatidylcholine with diacyl residue sum C38:3	PC aa C38:3	53478735
Phosphatidylcholine with diacyl residue sum C38:4	PC aa C38:4	53478701
Phosphatidylcholine with diacyl residue sum C38:5	PC aa C38:5	52923235
Phosphatidylcholine with diacyl residue sum C38:6	PC aa C38:6	24778898
Phosphatidylcholine with diacyl residue sum C40:1	PC aa C40:1	53479437
Phosphatidylcholine with diacyl residue sum C40:2	PC aa C40:2	53478745
Phosphatidylcholine with diacyl residue sum C40:3	PC aa C40:3	52923247
Phosphatidylcholine with diacyl residue sum C40:4	PC aa C40:4	53478881
Phosphatidylcholine with diacyl residue sum C40:6	PC aa C40:6	52922935
Phosphatidylcholine with diacyl residue sum C42:0	PC aa C42:0	24779162
Phosphatidylcholine with diacyl residue sum C42:1	PC aa C42:1	53479497
Phosphatidylcholine with diacyl residue sum C42:2	PC aa C42:2	52923201
Phosphatidylcholine with diacyl residue sum C42:4	PC aa C42:4	53478821

Phosphatidylcholine with diacyl residue sum C42:5	PC aa C42:5	52923265
Phosphatidylcholine with diacyl residue sum C42:6	PC aa C42:6	53479301
Phosphatidylcholine with acyl-alkyl residue sum C30:0	PC ae C30:0	24779275
Phosphatidylcholine with acyl-alkyl residue sum C30:1	PC ae C30:1	52923874
Phosphatidylcholine with acyl-alkyl residue sum C30:2	PC ae C30:2	53478639
Phosphatidylcholine with acyl-alkyl residue sum C32:1	PC ae C32:1	52923926
Phosphatidylcholine with acyl-alkyl residue sum C32:2	PC ae C32:2	52923928
Phosphatidylcholine with acyl-alkyl residue sum C34:0	PC ae C34:0	24779324
Phosphatidylcholine with acyl-alkyl residue sum C34:1	PC ae C34:1	53480705
Phosphatidylcholine with acyl-alkyl residue sum C34:2	PC ae C34:2	53478777
Phosphatidylcholine with acyl-alkyl residue sum C34:3	PC ae C34:3	24779386
Phosphatidylcholine with acyl-alkyl residue sum C36:0	PC ae C36:0	24779297
Phosphatidylcholine with acyl-alkyl residue sum C36:1	PC ae C36:1	53478887
Phosphatidylcholine with acyl-alkyl residue sum C36:2	PC ae C36:2	53478759
Phosphatidylcholine with acyl-alkyl residue sum C36:3	PC ae C36:3	53480743
Phosphatidylcholine with acyl-alkyl residue sum C36:4	PC ae C36:4	53478805
Phosphatidylcholine with acyl-alkyl residue sum C38:0	PC ae C38:0	24779329
Phosphatidylcholine with acyl-alkyl residue sum C38:1	PC ae C38:1	52923956
Phosphatidylcholine with acyl-alkyl residue sum C38:2	PC ae C38:2	53480811
Phosphatidylcholine with acyl-alkyl residue sum C38:3	PC ae C38:3	53478937
Phosphatidylcholine with acyl-alkyl residue sum C38:4	PC ae C38:4	53478939
Phosphatidylcholine with acyl-alkyl residue sum C38:5	PC ae C38:5	53480761
Phosphatidylcholine with acyl-alkyl residue sum C40:1	PC ae C40:1	53480717
Phosphatidylcholine with acyl-alkyl residue sum C40:2	PC ae C40:2	53480827
Phosphatidylcholine with acyl-alkyl residue sum C40:3	PC ae C40:3	53480829
Phosphatidylcholine with acyl-alkyl residue sum C40:4	PC ae C40:4	53479249
Phosphatidylcholine with acyl-alkyl residue sum C40:5	PC ae C40:5	53479269
Phosphatidylcholine with acyl-alkyl residue sum C40:6	PC ae C40:6	53480833
Phosphatidylcholine with acyl-alkyl residue sum C42:0	PC ae C42:0	24779354
Phosphatidylcholine with acyl-alkyl residue sum C42:1	PC ae C42:1	53480725
Phosphatidylcholine with acyl-alkyl residue sum C42:2	PC ae C42:2	53480841
Phosphatidylcholine with acyl-alkyl residue sum C42:3	PC ae C42:3	53480785
Phosphatidylcholine with acyl-alkyl residue sum C42:5	PC ae C42:5	6443119
Phosphatidylcholine with acyl-alkyl residue sum C44:3	PC ae C44:3	53481753
Phosphatidylcholine with acyl-alkyl residue sum C44:4	PC ae C44:4	53481761
Phosphatidylcholine with acyl-alkyl residue sum C44:5	PC ae C44:5	53481767
Phosphatidylcholine with acyl-alkyl residue sum C44:6	PC ae C44:6	53481755
Sphingomyelins		
Hydroxysphingomyelin with acyl residue sum C16:1	SM (OH) C16:1	53481780
Hydroxysphingomyelin with acyl residue sum C22:1	SM (OH) C22:1	53481785
Hydroxysphingomyelin with acyl residue sum C22:2	SM (OH) C22:2	53481787
Hydroxysphingomyelin with acyl residue sum C24:1	SM (OH) C24:1	53481791
Sphingomyelin with acyl residue sum C16:0	SM C16:0	5283590
Sphingomyelin with acyl residue sum C16:1	SM C16:1	53481781

Sphingomyelin with acyl residue sum C18:0	SM C18:0	5283588
Sphingomyelin with acyl residue sum C18:1	SM C18:1	6443882
Sphingomyelin with acyl residue sum C20:2	SM C20:2	44260124
Sphingomyelin with acyl residue sum C24:0	SM C24:0	5283595
Sphingomyelin with acyl residue sum C24:1	SM C24:1	44260126

Supplementary Table S2. Sums and ratios of metabolites quantified in bovine uterine luminal fluid samples, according to their biochemical classifications. Metabolites are categorized in amino acids (AA), biogenic amines (BA), acylcarnitines (AC), esters derived from dicarboxylic acids (DC), esters derived from hydroxylated acids (OH), Phosphatidylcholines (PC), Lysophosphatidylcholines (LysoPC), diacyl- (PC aa) or acyl-alkyl- (PC ae) phosphatidylcholines, saturated (SFA), monounsaturated (MUFA), polyunsaturated (PUFA) glycerophosphocholines, sphingomyelins (SM), hydroxysphingomyelins (SM-OH), and eicosanoids derived from the cycloxygenase (COX) and lipoxygenase (LOX) pathways.

**Biochemical classification**	**Calculations**
Total AA	Sum of the concentrations of Ala, Arg, Asn, Asp, Cit, Gln, Glu, Gly, Leu, Pro, Ser, Thr and Taurine
Non-essential AA	Sum of the concentrations of Ala, Asn, Asp, Gln, Gly, Pro, Ser and Taurine
Acidic AA	Sum of the concentrations of Asp and Glu
Small Neutral AA	Sum of Ala, Asn, Gly, Ser, Thr and Taurine
Osmotic-stress protection AA	Sum of Ala, Gln, Gly, Pro and Taurine
Glucogenic AA	Sum of Ala, Gly and Ser
Glutathione precursors AA	Sum of Glu and Gly
Total BA	Sum of Carnosine, Creatinine, Dopamine, Histamine, Putrescine, Sarcosine, SDMA, Spermidine, Spermine and t4-OH-Pro
Spermidine/Putrescine	Ratio of Spermidine to Putrescine
Spermine/Spermidine	Ratio of Spermine to Spermidine
Total Recoverable Amounts of AC	Sum of the concentrations of all acylcarnitines
Total short-chain AC	Sum of the concentrations of C2, C3, C3:1, C4, C4:1, C5 and C5:1
Total medium-chain AC	Sum of the concentrations of C6:1, C8, C9, C10, C10:1, C10:2, C12 and C12:1
Total long-chain AC	Sum of the concentrations of C14, C14:1, C14:2, C16, C16:1, C16:2, C18, C18:1 and C18:2
Acylcarnitine/Free carnitine	Ratio of Carnitine (C2) to Free carnitine (C0)
Total short-chain AC/Free carnitine	Ratio of total short-chain acylcarnitine (AC) to Free carnitine (C0)
CPT-I([C16+C18]/C0)	Ratio of [C16, C16-OH, C16:1, C16:1-OH, C16:2, C16:2-OH, C18, C18:1, C18:1-OH, C18:2] to Free carnitine (C0)
Total Esters derived from DC/Total AC	Ratio of Esters derived from DC to total AC
Esters derived from HO	Sum of the concentrations of C3-OH, C5-OH(C3-DC-M), C14:1-OH, C14:2-OH, C16-OH, C16:1-OH, C16:2-OH and C18:1-OH
Esters derived from DC	Sum of C3-DC(C4-OH), C5-DC(C6-OH), C5-M-DC, C5:1-DC, C7-DC and C12-DC
Total recoverable amounts of phospholipids	Sum of all phospholipids
Total recoverable amounts of LysoPC	Sum of the concentrations of lysoPC a C14:0, lysoPC a C16:0, lysoPC a C16:1, lysoPC a C17:0, lysoPC a C18:0, lysoPC a C18:1, lysoPC a C18:2, lysoPC a C20:3, lysoPC a C20:4, lysoPC a C24:0, lysoPC a C26:0, lysoPC a C26:1, lysoPC a C28:0 and lysoPC a C28:1

Total recoverable amounts of PC	Sum of the concentrations of PC aa C24:0, PC aa C26:0, PC aa C28:1, PC aa C30:0, PC aa C32:0, PC aa C32:1, PC aa C32:2, PC aa C32:3, PC aa C34:1, PC aa C34:2, PC aa C34:3, PC aa C34:4, PC aa C36:0, PC aa C36:1, PC aa C36:2, PC aa C36:3, PC aa C36:4, PC aa C36:5, PC aa C36:6, PC aa C38:0, PC aa C38:1, PC aa C38:3, PC aa C38:4, PC aa C38:5, PC aa C38:6, PC aa C40:1, PC aa C40:2, PC aa C40:3, PC aa C40:4, PC aa C40:6, PC aa C42:0, PC aa C42:1, PC aa C42:2, PC aa C42:4, PC aa C42:5, PC aa C42:6, PC ae C30:0, PC ae C30:1, PC ae C30:2, PC ae C32:1, PC ae C32:2, PC ae C34:0, PC ae C34:1, PC ae C34:2, PC ae C34:3, PC ae C36:0, PC ae C36:1, PC ae C36:2, PC ae C36:3, PC ae C36:4, PC ae C38:0, PC ae C38:1, PC ae C38:2, PC ae C38:3, PC ae C38:4, PC ae C38:5, PC ae C40:1, PC ae C40:2, PC ae C40:3, PC ae C40:4, PC ae C40:5, PC ae C40:6, PC ae C42:0, PC ae C42:1, PC ae C42:2, PC ae C42:3, PC ae C42:5, PC ae C44:3, PC ae C44:4, PC ae C44:5 and PC ae C44:6
Total LysoPC/Total PC[c]	Ratio of total LysoPC to total recoverable PC
Total PC aa	Sum of the concentrations of PC aa C24:0, PC aa C26:0, PC aa C28:1, PC aa C30:0, PC aa C32:0, PC aa C32:1, PC aa C32:2, PC aa C32:3, PC aa C34:1, PC aa C34:2, PC aa C34:3, PC aa C34:4, PC aa C36:0, PC aa C36:1, PC aa C36:2, PC aa C36:3, PC aa C36:4, PC aa C36:5, PC aa C36:6, PC aa C38:0, PC aa C38:1, PC aa C38:3, PC aa C38:4, PC aa C38:5, PC aa C38:6, PC aa C40:1, PC aa C40:2, PC aa C40:3, PC aa C40:4, PC aa C40:6, PC aa C42:0, PC aa C42:1, PC aa C42:2, PC aa C42:4, PC aa C42:5 and PC aa C42:6
Total PC ae	Sum of the concentrations of PC ae C30:0, PC ae C30:1, PC ae C30:2, PC ae C32:1, PC ae C32:2, PC ae C34:0, PC ae C34:1, PC ae C34:2, PC ae C34:3, PC ae C36:0, PC ae C36:1, PC ae C36:2, PC ae C36:3, PC ae C36:4, PC ae C38:0, PC ae C38:1, PC ae C38:2, PC ae C38:3, PC ae C38:4, PC ae C38:5, PC ae C40:1, PC ae C40:2, PC ae C40:3, PC ae C40:4, PC ae C40:5, PC ae C40:6, PC ae C42:0, PC ae C42:1, PC ae C42:2, PC ae C42:3, PC ae C42:5, PC ae C44:3, PC ae C44:4, PC ae C44:5 and PC ae C44:6
Total MUFA (PC)	Sum of the concentrations of PC aa C28:1, PC aa C32:1, PC aa C34:1, PC aa C36:1, PC aa C38;1, PC aa C40:1, PC aa C42:1, PC ae C30:1, PC ae C32:1, PC ae C34:1, PC ae C36:1, PC ae C38:1, PC ae C40:1 and PC ae C42:1
Total PUFA (PC)	Sum of the concentrations of PC aa C32:2, PC aa C32:3, PC aa C34:2, PC aa C 34:3, PC aa 34:4, PC aa C36:2, PC aa C36:3, PC aa C36:4, PC aa C36:5, PC aa C36:6, PC aa C38:3, PC aa C38:4, PC aa C38:5, PC aa C38:6, PC aa C40:2, PC aa C40:3, PC aa C40:4, PC aa C40:6, PC aa C42:2, PC aa C42:4, PC aa C42:5, PC aa C42:6, PC ae C30:2, PC ae C32:2, PC ae C34:2, PC ae C34:3, PC ae C36:2, PC ae C36:3, PC ae C36:4, PC ae C38:2, PC ae C38:3, PC ae C38:4, PC ae C38:5, PC ae C40:2, PC ae C40:3, PC ae C40:4, PC ae C40:5, PC ae C40:6, PC ae C42:2, PC ae C42:3, PC ae C42:5, PC ae C44:3, PC ae C44:4, PC ae C44:5 and PC ae C44:6
Total SFA (PC)	Sum of the concentrations of PC aa C24:0, PC aa C26:0, PC aa C30:0, PC aa C32:0, PC aa C36:0, PC aa C38:0, PC aa C42:0, PC ae C30:0, PC ae C34:0, PC ae C36:0, PC ae C38:0 and PC ae C42:0
MUFA (PC)/SFA (PC)[d]	Ratio of monounsaturated fatty acids (MUFA) to saturated fatty acids (SFA)
PUFA (PC)/MUFA (PC)[d]	Ratio of polyunsaturated fatty acids (PUFA) to monounsaturated fatty acids (MUFA)
PUFA (PC)/SFA (PC)[d]	Ratio of polyunsaturated fatty acids (PUFA) to saturated fatty acids (SFA)
Total SM	Sum of all sphingomyelins (SM)
Total SM-OH	Sum of the concentrations of SM (OH) C16:1, SM (OH) C22:1, SM (OH) C22:2, SM (OH) C24:1
Ratio SM/SM-OH	Ratio of total recoverable sphingomyelins (SM) to hydroxysphingomyelins (SM-OH)
Total unsaturated SM	Sum of the concentrations of SM C16:1, SM C18:1 and SM C24:1

Total saturated SM	Sum of the concentrations of SM C16:0, SM C18:0 and SM C24:0
Hexoses	Sum of the concentrations of Glucose; Aldohexose; L-Allopyranose; D-Allose; D-Allopyranose; D-Allose; D-Altropyranose; D-Glucopyranose; alpha-D-Glucopyranose; beta-D-Glucopyranose; D-Mannopyranose; alpha-D; Mannopyranose; L-Gulopyranose; D-Gulopyranose; D-Idopyranose; Alpha-L-Galactopyranose; alpha-D-Galactopyranose; beta-D-Galactopyranose; D-Talose; D-Talopyranose; Ketohexose; D-Psicopyranose; L-Fructofuranose; D-Fructose; D-Fructofuranose; L-Sorbopyranose; D-Sorbopyranose; D-Tagatose; D-Tagatopyranose
COX pathway	Sum of the concentrations of 6-keto-PGF1a and PGF2a
LOX pathway	Sum of the concentrations of 12S-HETE, 15S-HETE and 13S-HODE

Supplementary Table S3. Bovine specific oligonucleotide forward and reverse primer sequences (5'-3') and PCR product length.

Gene symbol	Gene	GenBank accession	Forward and Reverse sequences	Amplicon size (bp)
ALOX5	arachidonate 5-lipoxygenase	NM_001192792.2	5' CAAGCAGCACAGACGCAAAGAACT<br>3'AAGTCCTTGTGGCATTTGGCATCG	108
ALOX5AP	arachidonate 5-lipoxygenase activating protein	NM_001076293	5' ACACTGCCAACCAGAACTGTGT<br>3' CTGCCTCACGAACAGGTACATC	125
ALOX12	arachidonate 12-lipoxygenase	NM_001192336.1	5' GTCCTAACCCAGCCATGTTT<br>3' GCCCAGTCAGTCTTCAGTTT	163
ALOX15B	arachidonate 15-lipoxygenase, type B	NM_001205703.1	5' TCTTCAAGCTGCTGATCCCTCACA<br>3' ATATCATCAGGCAGACACAGGGCA	187
SLC6A9	solute carrier family 6, member 9	NM_001242343.1	5' TGTTCAAAGGTGTGGGCTAC<br>3' GGCGTGTTCCAAGGGTTATT	151
LPL	lipoprotein lipase	NM_001075120.1	5' AACCGGACTCCAACGTCATC<br>3' TTCATCCGCCATCCAGTTC	128
PPARG	peroxisome proliferator activated receptor gamma	NM_181024.2	5' AAGCCCTTTGGTGACTTTATGG<br>3' GGCGGTCTCCACTGAGAATAAT	121
RXRA	retinoid X receptor alpha	NM_001304343.1	5' AAGATGCGGGACATGCAGAT<br>3' CAGCTTGGCGAACCTTCCT	189
ACTB	actin, beta	NM_173979.3	5' GGATGAGGCTCAGAGCAAGAGA<br>3' TCGTCCCAGTTGGTGACGAT	76
GAPDH	glyceraldehyde-3-phosphate dehydrogenase	NM_001034034.2	5' GCCATCAATGACCCCTTCAT<br>3' TGCCGTGGGTGGAATCA	68
PPIA	peptidylprolyl isomerase A	NM_178320.2	5' GCCATGGAGCGCTTTGG<br>3' CCACAGTCAGCAATGGTGATCT	63

Supplementary Table S4. Amino acids and biogenic amines concentration in uterine luminal fluid from Control (Con) and Pregnant (Preg) cows. Values are expressed as nmol/cm^2 of endometrial area; mean ± SEM.

Metabolite	Group		P value	FDR significance[a]	Log2 Fold-change[b]
	Con (n = 8)	Preg (n = 10)			
Amino acids					
Alanine	1236.58 ± 148.21	1143.85 ± 54.05	0.70	n.s.	-0.11
Arginine	39.02 ± 11.07	18.71 ± 2.36	0.14	n.s.	-1.06
Asparagine	74.30 ± 4.37	67.21 ± 2.38	0.47	n.s.	-0.14
Aspartate	636.89 ± 66.30	578.99 ± 27.41	0.61	n.s.	-0.14
Citrulline	33.57 ± 6.22	27.16 ± 1.95	0.48	n.s.	-0.31
Glutamate	5538.84 ± 367.80	5179.54 ± 142.01	0.56	n.s.	-0.10
Glutamine	1008.70 ± 123.14	1028.15 ± 43.33	0.92	n.s.	0.03
**Glycine**	5099.80 ± 506.62	3588.41 ± 123.76	0.03	*	-0.51
Leucine	69.83 ± 26.46	29.77 ± 4.47	0.16	n.s.	-1.22
Proline	250.44 ± 31.07	172.26 ± 10.60	0.09	n.s.	-0.54
Serine	202.29 ± 33.24	145.71 ± 10.96	0.25	n.s.	-0.47
Threonine	218.88 ± 23.40	211.43 ± 6.94	0.83	n.s.	-0.04
Biogenic amines					
Carnosine	51.65 ± 7.73	40.12 ± 1.73	0.23	n.s.	-0.36
Creatinine	156.23 ± 14.69	141.52 ± 5.20	0.55	n.s.	-0.14
Dopamine	2.52 ± 0.58	2.19 ± 0.10	0.60	n.s.	-0.20
Histamine	81.11 ± 21.16	45.23 ± 3.57	0.13	n.s.	-0.84
Putrescine	349.09 ± 52.27	304.01 ± 14.13	0.51	n.s.	-0.20
**Sarcosine**	515.78 ± 54.96	311.97 ± 10.02	0.005	**	-0.74
Spermidine	61.68 ± 6.78	46.43 ± 1.57	0.08	n.s.	-0.42
Spermine	44.96 ± 10.64	30.52 ± 2.29	0.26	n.s.	-0.56
SDMA	0.58 ± 0.18	0.66 ± 0.07	0.81	n.s.	0.16
Taurine	4074.73 ± 369.12	3418.52 ± 122.18	0.23	n.s.	-0.25
Trans-4-Hydroxyproline	46.59 ± 5.67	44.42 ± 2.03	0.81	n.s.	-0.07

Metabolites in bold were different between Con and Preg group by ANOVA followed by FDR correction.
[a]Statistical analyses were carried out by one-way ANOVA followed by FDR correction for multiple comparisons. Magnitude of effect is indicated by: ** $P \leq 0.01$; * $P \leq 0.05$; n.s. $P > 0.05$.
[b]Data are represented as fold-change of the metabolite concentration between Preg and Con groups.

Supplementary Table S5. Carnitine and acylcarnitines concentration in uterine luminal fluid from Control (Con) and Pregnant (Preg) cows. Values are expressed as nmol/cm^2 of endometrial area; mean ± SEM.

Metabolite	Group		P value	FDR significance[a]	Log2 Fold-change[b]
	Con (n = 8)	Preg (n = 10)			
Carnitine free (C0)	99.14 ± 10.28	100.51 ± 4.03	0.93	n.s.	0.01
Short-chain acylcarnitine					
C2	63.18 ± 8.10	59.72 ± 2.51	0.76	n.s.	-0.07
C3	2.50 ± 0.28	2.50 ± 0.12	0.99	n.s.	0.00
C3:1	0.45 ± 0.03	0.39 ± 0.01	0.18	n.s.	-0.20
C4	7.61 ± 1.43	9.47 ± 0.60	0.61	n.s.	0.31
C4:1	0.81 ± 0.05	0.75 ± 0.02	0.40	n.s.	-0.12
C5	1.71 ± 0.18	1.67 ± 0.07	0.89	n.s.	-0.03
C5:1	1.14 ± 0.10	1.17 ± 0.03	0.85	n.s.	0.03
Medium-chain acylcarnitine					
C6	1.83 ± 0.12	1.51 ± 0.03	0.04	n.s.	-0.29
C6:1	0.91 ± 0.07	0.91 ± 0.02	0.97	n.s.	0.00
C8	2.70 ± 0.18	2.46 ± 0.05	0.30	n.s.	-0.14
C9	0.60 ± 0.05	0.58 ± 0.01	0.73	n.s.	-0.04
C10	2.41 ± 0.18	2.02 ± 0.03	0.06	n.s.	-0.25
C10:1	1.83 ± 0.17	1.72 ± 0.04	0.60	n.s.	-0.09
C10:2	0.81 ± 0.06	0.76 ± 0.01	0.48	n.s.	-0.09
C12	1.91 ± 0.12	1.88 ± 0.03	0.87	n.s.	-0.01
C12:1	1.59 ± 0.15	1.48 ± 0.03	0.50	n.s.	-0.10
Long-chain acylcarnitine					
C14	0.71 ± 0.07	0.63 ± 0.02	0.36	n.s.	-0.17
C14:1	0.20 ± 0.02	0.19 ± 0.01	0.61	n.s.	-0.10
C14:2	1.41 ± 0.08	1.36 ± 0.03	0.69	n.s.	-0.06
C16	0.44 ± 0.05	0.37 ± 0.01	0.21	n.s.	-0.23
C16:1	0.60 ± 0.05	0.51 ± 0.01	0.15	n.s.	-0.23
C16:2	0.26 ± 0.03	0.25 ± 0.01	0.89	n.s.	-0.03
C18	0.41 ± 0.04	0.30 ± 0.01	0.04	n.s.	-0.47
C18:1	0.75 ± 0.09	0.55 ± 0.02	0.05	n.s.	-0.45
C18:2	0.28 ± 0.03	0.29 ± 0.01	0.86	n.s.	0.04
Esters derived from dicarboxylic acids					
C3-DC	1.30 ± 0.10	1.22 ± 0.03	0.60	n.s.	-0.09
C5:1-DC	0.72 ± 0.05	0.63 ± 0.01	0.17	n.s.	-0.22
C5-DC	0.40 ± 0.02	0.38 ± 0.01	0.30	n.s.	-0.10
C5-M-DC	1.02 ± 0.09	0.95 ± 0.02	0.52	n.s.	-0.10
C7-DC	0.35 ± 0.02	0.32 ± 0.004	0.22	n.s.	-0.12
C12-DC	3.02 ± 0.21	2.88 ± 0.04	0.56	n.s.	-0.06
Esters derived from hydroxylated acids					
**C3-OH**	0.60 ± 0.03	0.47 ± 0.01	0.005	**	-0.36
**C5-OH**	1.51 ± 0.08	1.14 ± 0.03	0.02	*	-0.40
C14:1-OH	0.32 ± 0.03	0.29 ± 0.01	0.30	n.s.	-0.17
C14:2-OH	0.37 ± 0.03	0.36 ± 0.01	0.79	n.s.	-0.04

**C16-OH**	0.33 ± 0.02	0.27 ± 0.004	0.03	*	-0.27
C16:1-OH	0.30 ± 0.02	0.26 ± 0.01	0.20	n.s.	-0.20
C16:2-OH	0.48 ± 0.04	0.54 ± 0.01	0.33	n.s.	0.16
C18:1-OH	0.63 ± 0.04	0.65 ± 0.01	0.73	n.s.	0.03

Metabolites in bold were different between Con and Preg group by ANOVA followed by FDR correction.
[a]Statistical analyses were carried out by one-way ANOVA followed by FDR correction for multiple comparisons. Magnitude of effect is indicated by: ** $P \leq 0.01$; * $P \leq 0.05$; n.s. $P > 0.05$.
[b]Data are represented as fold-change of the metabolite concentration between Preg and Con groups.

Supplementary Table S6. Phosphatidylcholines (PC) and Lysophosphatidylcholines (LysoPC) concentration in uterine luminal fluid from Control (Con) and Pregnant (Preg) cows. Values are expressed as nmol/cm^2 of endometrial area; mean ± SEM.

Metabolite	Group		P value	FDR significance[a]	Log2 Fold-change[b]
	Con (n = 8)	Preg (n = 10)			
Lysophosphatidylcholines					
lysoPC a C14:0	241.28 ± 16.48	243.82 ± 3.64	0.90	n.s.	0.01
lysoPC a C16:0	3.77 ± 0.41	3.29 ± 0.12	0.42	n.s.	-0.20
lysoPC a C16:1	1.68 ± 0.09	1.88 ± 0.03	0.11	n.s.	0.16
**lysoPC a C17:0**	0.91 ± 0.06	0.64 ± 0.03	0.02	*	-0.49
lysoPC a C18:0	5.47 ± 0.15	5.56 ± 0.11	0.85	n.s.	0.03
lysoPC a C18:1	2.91 ± 0.32	3.02 ± 0.09	0.82	n.s.	0.06
**lysoPC a C18:2**	1.88 ± 0.23	1.27 ± 0.04	0.02	*	-0.58
lysoPC a C20:3	2.35 ± 0.26	1.73 ± 0.04	0.05	n.s.	-0.43
lysoPC a C20:4	0.47 ± 0.06	0.56 ± 0.02	0.35	n.s.	0.26
lysoPC a C24:0	15.11 ± 1.01	14.50 ± 0.18	0.59	n.s.	-0.06
lysoPC a C26:0	0.45 ± 0.05	0.57 ± 0.02	0.14	n.s.	0.33
lysoPC a C26:1	0.27 ± 0.05	0.30 ± 0.01	0.66	n.s.	0.15
lysoPC a C28:0	2.17 ± 0.17	2.13 ± 0.05	0.87	n.s.	-0.03
lysoPC a C28:1	0.46 ± 0.05	0.38 ± 0.03	0.41	n.s.	-0.27
Diacyl-phosphatidylcholines					
PC aa C24:0	0.54 ± 0.03	0.53 ± 0.01	0.74	n.s.	-0.03
PC aa C26:0	17.63 ± 1.28	16.65 ± 0.23	0.50	n.s.	-0.09
PC aa C28:1	0.39 ± 0.05	0.36 ± 0.01	0.51	n.s.	-0.14
PC aa C30:0	3.25 ± 0.23	2.99 ± 0.06	0.37	n.s.	-0.12
PC aa C32:0	1.58 ± 0.17	1.28 ± 0.05	0.27	n.s.	-0.30
PC aa C32:1	0.92 ± 0.18	0.66 ± 0.03	0.18	n.s.	-0.49
PC aa C32:2	0.98 ± 0.23	0.63 ± 0.04	0.19	n.s.	-0.62
PC aa C32:3	0.68 ± 0.18	0.32 ± 0.02	0.06	n.s.	-1.09
PC aa C34:1	15.79 ± 3.24	10.29 ± 0.49	0.11	n.s.	-0.62
PC aa C34:2	3.20 ± 0.48	2.07 ± 0.10	0.06	n.s.	-0.62
PC aa C34:3	0.80 ± 0.17	0.42 ± 0.02	0.04	n.s.	-0.92
PC aa C34:4	0.46 ± 0.11	0.25 ± 0.02	0.08	n.s.	-0.89
**PC aa C36:0**	8.89 ± 0.63	7.25 ± 0.11	0.02	*	-0.29
PC aa C36:1	8.12 ± 1.40	5.39 ± 0.21	0.07	n.s.	-0.60
PC aa C36:2	6.95 ± 1.01	4.54 ± 0.19	0.04	n.s.	-0.62
**PC aa C36:3**	2.51 ± 0.42	1.43 ± 0.06	0.02	*	-0.81
PC aa C36:4	1.87 ± 0.38	1.27 ± 0.07	0.16	n.s.	-0.56
**PC aa C36:5**	0.58 ± 0.09	0.32 ± 0.01	0.02	*	-0.86
PC aa C36:6	0.31 ± 0.09	0.21 ± 0.01	0.27	n.s.	-0.58
PC aa C38:0	0.67 ± 0.09	0.60 ± 0.01	0.40	n.s.	-0.17
PC aa C38:1	0.43 ± 0.11	0.33 ± 0.02	0.37	n.s.	-0.40
PC aa C38:3	1.41 ± 0.30	0.77 ± 0.04	0.05	n.s.	-0.86
PC aa C38:4	2.27 ± 0.43	1.58 ± 0.07	0.12	n.s.	-0.51
PC aa C38:5	1.49 ± 0.29	1.17 ± 0.06	0.34	n.s.	-0.34

PC aa C38:6	0.71 ± 0.14	0.66 ± 0.03	0.76	n.s.	-0.10
PC aa C40:1	6.18 ± 0.41	6.35 ± 0.10	0.74	n.s.	0.04
PC aa C40:2	0.16 ± 0.03	0.16 ± 0.01	0.84	n.s.	0.06
PC aa C40:3	0.14 ± 0.03	0.10 ± 0.01	0.20	n.s.	-0.47
PC aa C40:4	0.60 ± 0.17	0.38 ± 0.02	0.22	n.s.	-0.69
PC aa C40:6	6.38 ± 0.49	5.90 ± 0.10	0.39	n.s.	-0.12
PC aa C42:0	0.87 ± 0.06	0.84 ± 0.02	0.79	n.s.	-0.04
PC aa C42:1	0.15 ± 0.03	0.17 ± 0.01	0.54	n.s.	0.20
PC aa C42:2	1.89 ± 0.13	1.63 ± 0.03	0.09	n.s.	-0.22
PC aa C42:4	0.22 ± 0.03	0.17 ± 0.01	0.14	n.s.	-0.38
PC aa C42:5	0.19 ± 0.05	0.15 ± 0.01	0.53	n.s.	-0.30
PC aa C42:6	1.93 ± 0.18	1.71 ± 0.04	0.34	n.s.	-0.17
Acyl-alkyl-phosphatidylcholines					
PC ae C30:0	1.91 ± 0.18	1.56 ± 0.03	0.10	n.s.	-0.29
PC ae C30:1	0.38 ± 0.12	0.24 ± 0.03	0.36	n.s.	-0.69
PC ae C30:2	7.21 ± 0.52	6.29 ± 0.09	0.12	n.s.	-0.20
PC ae C32:1	0.47 ± 0.14	0.36 ± 0.02	0.42	n.s.	-0.40
PC ae C32:2	0.58 ± 0.12	0.40 ± 0.02	0.15	n.s.	-0.54
PC ae C34:0	0.66 ± 0.12	0.58 ± 0.02	0.55	n.s.	-0.18
PC ae C34:1	1.85 ± 0.31	1.34 ± 0.07	0.19	n.s.	-0.47
**PC ae C34:2**	1.32 ± 0.22	0.70 ± 0.04	0.02	*	-0.92
PC ae C34:3	0.67 ± 0.13	0.38 ± 0.02	0.05	n.s.	-0.81
PC ae C36:0	3.15 ± 0.21	2.74 ± 0.05	0.13	n.s.	-0.20
PC ae C36:1	2.18 ± 0.31	1.47 ± 0.05	0.05	n.s.	-0.58
**PC ae C36:2**	1.53 ± 0.13	1.12 ± 0.02	0.01	*	-0.45
PC ae C36:3	0.31 ± 0.05	0.21 ± 0.01	0.16	n.s.	-0.56
PC ae C36:4	0.73 ± 0.10	0.54 ± 0.02	0.07	n.s.	-0.43
PC ae C38:0	1.94 ± 0.19	1.72 ± 0.03	0.29	n.s.	-0.17
**PC ae C38:1**	0.32 ± 0.05	0.16 ± 0.01	0.01	*	-0.97
**PC ae C38:2**	0.31 ± 0.03	0.19 ± 0.01	0.03	*	-0.69
**PC ae C38:3**	0.30 ± 0.03	0.17 ± 0.01	0.01	*	-0.79
PC ae C38:4	0.79 ± 0.08	0.60 ± 0.02	0.04	n.s.	-0.40
PC ae C38:5	0.48 ± 0.10	0.28 ± 0.01	0.06	n.s.	-0.76
**PC ae C40:1**	0.32 ± 0.02	0.20 ± 0.01	0.002	**	-0.64
PC ae C40:2	0.15 ± 0.02	0.14 ± 0.00	0.80	n.s.	-0.06
PC ae C40:3	0.09 ± 0.04	0.09 ± 0.01	0.97	n.s.	0.01
PC ae C40:4	1.17 ± 0.11	0.98 ± 0.02	0.14	n.s.	-0.25
PC ae C40:5	0.18 ± 0.04	0.12 ± 0.01	0.21	n.s.	-0.56
**PC ae C40:6**	0.27 ± 0.03	0.17 ± 0.01	0.03	*	-0.64
PC ae C42:0	14.45 ± 0.85	14.32 ± 0.22	0.90	n.s.	-0.01
PC ae C42:1	1.41 ± 0.13	1.30 ± 0.03	0.49	n.s.	-0.12
**PC ae C42:2**	0.27 ± 0.04	0.17 ± 0.01	0.03	*	-0.69
PC ae C42:3	0.13 ± 0.02	0.09 ± 0.01	0.15	n.s.	-0.51
PC ae C42:5	20.61 ± 1.16	21.71 ± 0.28	0.46	n.s.	0.07

PC ae C44:3	0.57 ± 0.04	0.64 ± 0.01	0.21	n.s.	0.15
PC ae C44:4	1.91 ± 0.11	1.76 ± 0.03	0.28	n.s.	-0.12
PC ae C44:5	1.05 ± 0.08	0.97 ± 0.02	0.43	n.s.	-0.12
**PC ae C44:6**	0.48 ± 0.02	0.35 ± 0.01	0.003	**	-0.47

Metabolites in bold were different between Con and Preg group by ANOVA followed by FDR correction.
[a]Statistical analyses were carried out by one-way ANOVA followed by FDR correction for multiple comparisons. Magnitude of effect is indicated by: ** $P \leq 0.01$; * $P \leq 0.05$; n.s. $P > 0.05$.
[b]Data are represented as fold-change of the metabolite concentration between Preg and Con groups.

Supplementary Table S7. Sphingomyelins (SM) concentration in uterine luminal fluid from Control (Con) and Pregnant (Preg) cows. Values are expressed as nmol/cm^2 of endometrial area; mean ± SEM.

**Metabolite**	**Group**		**P value**	**FDR significance**[a]	**Log2 Fold-change**[b]
	**Con (n = 8)**	**Preg (n = 10)**			
Sphingomyelins					
SM C16:0	23.14 ± 4.25	17.86 ± 0.92	0.31	n.s.	-0.38
SM C16:1	1.23 ± 0.32	0.80 ± 0.04	0.17	n.s.	-0.62
SM C18:0	4.30 ± 0.83	3.17 ± 0.17	0.24	n.s.	-0.43
SM C18:1	0.70 ± 0.12	0.52 ± 0.03	0.27	n.s.	-0.42
SM C24:0	4.03 ± 0.55	2.76 ± 0.10	0.05	n.s.	-0.56
SM C24:1	3.47 ± 0.55	2.99 ± 0.15	0.53	n.s.	-0.22
Hydroxysphingomyelins					
SM(OH) C16:1	1.94 ± 0.24	1.28 ± 0.06	0.04	n.s.	-0.60
SM(OH) C22:1	2.10 ± 0.42	1.23 ± 0.06	0.06	n.s.	-0.76
SM(OH) C22:2	0.52 ± 0.18	0.38 ± 0.01	0.35	n.s.	-0.43
SM(OH) C24:1	0.33 ± 0.05	0.29 ± 0.02	0.62	n.s.	-0.18

[a]Statistical analyses were carried out by one-way ANOVA followed by FDR correction for multiple comparisons. Magnitude of effect is indicated by: ** $P \leq 0.01$; * $P \leq 0.05$; n.s. $P > 0.05$.
[b]Data are represented as fold-change of the metabolite concentration between Preg and Con groups.

Supplementary Table S8. Hexoses concentration in uterine luminal fluid from Control (Con) and Pregnant (Preg) cows. Values are expressed as nmol/cm^2 of endometrial area; mean ± SEM.

Metabolite	Group		P value	FDR significance[a]	Log2 Fold-change[b]
	Con (n = 8)	Preg (n = 10)			
Hexoses[c]	337,367.78 ± 20441.23	331,581.47 ± 5435.96	0.83	n.s.	-0.02

[a]Statistical analyses were carried out by one-way ANOVA followed by FDR correction for multiple comparisons. Magnitude of effect is indicated by: ** $P \leq 0.01$; * $P \leq 0.05$; n.s. $P > 0.05$.

[b]Data are represented as fold-change of the metabolite concentration between Preg and Con groups.

[c]Sum of the concentrations of the following hexoses: Glucose; Aldohexose; L-Allopyranose; D-Allose; D-Allopyranose; D-Allose; D-Altropyranose; D-Glucopyranose; alpha-D-Glucopyranose; beta-D-Glucopyranose; D-Mannopyranose; alpha-D; Mannopyranose; L-Gulopyranose; D-Gulopyranose; D-Idopyranose; Alpha-L-Galactopyranose; alpha-D-Galactopyranose; beta-D-Galactopyranose; D-Talose; D-Talopyranose; Ketohexose; D-Psicopyranose; L-Fructofuranose; D-Fructose; D-Fructofuranose; L-Sorbopyranose; D-Sorbopyranose; D-Tagatose; D-Tagatopyranose.
